# Predicting the distribution of *Ixodes ricinus* in Europe: integrating microclimatic factors into ecological niche models

**DOI:** 10.1017/S003118202400132X

**Published:** 2024-08

**Authors:** Arda Cem Kuyucu, Olcay Hekimoglu

**Affiliations:** Biology Department, Hacettepe University, Ankara, Turkey

**Keywords:** climate change, ecological niche modelling, *Ixodes ricinus*, Maxent, microclimate, ticks

## Abstract

*Ixodes ricinus*, commonly known as the castor bean tick and sheep tick, is a significant vector of various diseases, such as tick-borne encephalitis and Lyme borreliosis. Owing to climate change, the distribution and activity of *I. ricinus* are expected to increase, leading to an increase in the number of diseases transmitted by this species. Most distribution models and ecological niche models utilize macroclimate datasets such as WorldClim or CHELSA to map the distribution of disease-transmitting ticks. However, microclimatic factors are crucial for the activity and survival of small arthropods. In this study, an ecological niche modelling approach was used to assess the climatic suitability of *I. ricinus* using both microclimatic and macroclimatic parameters. A Mixed model was built by combining parameters from the Soiltemp (microclimate) and Wordclim (macroclimate) databases, whereas a Macroclimate model was built with the CHELSA dataset. Additionally, future suitabilities were projected *via* the macroclimate model under the SSP3-7.0 and SSP5-8.5 scenarios. Macroclimate and Mixed models showed similar distributions, confirming the current distribution of *I. ricinus*. The most important climatic factors were seasonality, annual temperature range, humidity and precipitation. Future projections suggest significant expansion in northern and eastern Europe, with notable declines in southern Europe.

## Graphical abstract

**Figure:**
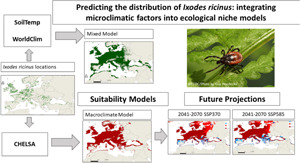

## Introduction

As anthropogenic climate change becomes increasingly apparent through rising mean annual temperatures and the significant increase in both the frequency and magnitude of extreme weather events, investigations of the relationships between vector populations and climate have garnered increased prominence (Kilpatrick and Randolph, 2012; Dantas-Torres, 2015; Semenza and Suk, 2018; Rocklöv and Dubrow, 2020; Gilbert, 2021). Consequently, studying how vectors interact with their climatic environment, with a focus on mechanistic and correlative models, has gained additional importance for human health and welfare (Estrada-Peña, 2008; Li *et al*., 2012; Zhao *et al*., 2021).

*Ixodes ricinus* is one of the most important tick species in Europe because of its role as a vector for Lyme disease and tick-borne encephalitis (Jaenson and Lindgren, 2011; Ostfeld and Brunner, 2015; Estrada-Peña *et al*., 2016). The abiotic factors that affect the abundance and distribution of *I. ricinus* have long been studied. Specifically, humidity and vegetation litter layers have been noted as the most important requirements for the survival and host-seeking activity of the species (Knülle and Rudolph, 1982; Gray *et al*., 2016; Van Gestel *et al*., 2022). Geographical variation in temperature is another significant factor that has been suggested to influence the timing of host-seeking activity and molting to the next life stage (Jaenson and Lindgren, 2011; Gilbert *et al*., 2014). As a result of global warming, changes in precipitation patterns and increasing temperatures could increase the mortality rate of *I. ricinus*, particularly due to the potential increase in droughts and cold periods (Dautel *et al*., 2016). Conversely, the occurrence of milder winters could create new distribution areas with altitudinal shifts, such as mountainous regions and higher latitudes in Europe (Materna *et al*., 2008; Jaenson and Lindgren, 2011; Martello *et al*., 2014; Hvidsten *et al*., 2020).

Ecological niche modelling (ENM) is a fundamental correlative method for investigating the distribution of disease transmission vectors, including ticks (Raghavan *et al*., 2020; Alkishe *et al*., 2021; Moo-Llanes *et al*., 2021). ENMs combine the locations of species with environmental variables to construct a representative multidimensional niche of the species (Warren and Seifert, 2011; Peterson and Soberón, 2012). In addition to predicting the current potential niches of the species, ENMs are utilized to project possible future distributions under global circulation model (GCM) scenarios (Carvalho *et al*., 2017; Aguilar-Domínguez *et al*., 2021; Alkishe and Peterson, 2022). Although most distribution models use macroclimatic parameters gathered from standardized weather stations, the conditions of the microclimate can differ greatly from those of the macroclimate (Maclean *et al*., 2021; Marcin *et al*., 2021); recently, there have been significant advances in including microclimatic parameters in distribution modelling (Lembrechts *et al*., 2019; Stark and Fridley, 2022). The microclimate might be more decisive than the macroclimate for small arthropods such as ticks because they are much more exposed to conditions close to the ground and under litter, and conditions in the microclimate could greatly affect the survival and activity of ticks (Randolph and Storey, 1999; Lauterbach *et al*., 2013; Boehnke *et al*., 2017; Volk *et al*., 2022).

Several modelling studies have suggested possible changes in the distribution areas of *I. ricinus* in the future, which could increase the risk of dissemination of related diseases. The common conclusion drawn from these studies is that the range has expanded to a large part of Europe, North Africa and the Middle East (Porretta *et al*., 2013; Alkishe *et al*., 2017; Cunze *et al*., 2022). However, a significant portion of the studies included specimens from North Africa, which were later identified as *Ixodes inopinatus* (Estrada-Peña *et al*., 2014). Therefore, reevaluating previous occurrence records is extremely necessary. Additionally, occurrence records from countries such as Turkey, where *I. ricinus* was observed but generally was neglected in these analyses, will provide important information about the current and future distributions of the species. Additionally, most previous studies except Cunze *et al*. (2022) on *I. ricinus* have used CMIP5 and earlier GCMs to predict possible future climatic suitabilities, and updated projections that use the latest GCMs will provide better predictions.

The primary goal of this study was to predict climatically favourable areas for *I. ricinus* under both current and future climate conditions *via* the Maxent approach by incorporating microclimatic and macroclimatic variables. Tick presence data were gathered from the current literature, and a Macroclimate suitability model was built using the CHELSA bioclimatic dataset, whereas a Mixed (microclimate and macroclimate) suitability model was built by combining Soiltemp and WorldClim datasets. The second aim was to estimate the possible future distributions under projected GCMs using suitability models. To this end, SSP3-7.0 and SSP5-8.5 GCMs for the 2011–2040, 2041–2070 and 2071–2100 periods, respectively, were used to carry out future projections with the Macroclimate model. The results of this study will be informative for evaluating the risk of tick-borne diseases and predicting future threats posed by emerging diseases transmitted by *I. ricinus*.

## Materials and methods

### Species distribution data

To infer the current and future distributions of *I. ricinus*, various resources have been utilized to gather occurrence data: VectorMap (www.vectormap.org), the Global Biodiversity Information Facility (www.gbif.org) and scientific literature (Boehnke *et al*., 2015; Estrada-Pena and De La Fuente, 2016; Krawczyk *et al*., 2022). Occurrence points from Turkey were primarily derived from Hekimoğlu (2022), who utilized both morphological and molecular methods for sample identification. Additionally, field sampling data from 2023 (*n* = 2, İstanbul) were included in the final local dataset (*n* = 19).

The raw records were cleaned and refined by excluding localities (1) where tick collection was conducted from birds, as the presence of ticks on birds does not necessarily indicate that ticks establish populations in those areas; (2) samples from North Africa, as these have been redetermined as *I. inopinatus* (Rollins *et al*., 2023), whereas *I. inopinatus* could be distributed in southern Spain and Portugal, the absence of clear evidence distinguishing them from *I. ricinus* led us to retain the geographical points of these countries in the analyses. (3) Locations from the Mediterranean and Aegean regions of Turkey were excluded because of a lack of confirmation in the scientific literature. Following these steps, a preliminary set of 4668 geographic location points was obtained.

### Environmental variables

Microclimatic predictors from the Soiltemp (available at https://zenodo.org/records/7134169) dataset were used to build the first model (Lembrechts *et al*., 2020) with 30 arcsec (~1 km) resolution. The Soiltemp dataset includes bioclimatic temperature variables similar to those of the WorldClim and CHELSA datasets. However, unlike these datasets, which use interpolated data from standardized weather stations, the environmental data in the Soiltemp dataset are interpolated from both observational and experimental data gathered from dataloggers placed in microclimates (in soil) worldwide. Another advantage of Soiltemp is that the most intense data gathering was carried out in Europe, the main distributional area of *I. ricinus*. As the Soiltemp data included only temperature-related parameters (the first 11 bioclimatic variables), they were combined with precipitation parameters (bioclimatic vars 11–17), vapour pressure and surface radiation parameters from the WorldClim dataset (Fick and Hijmans, 2017) available at https://www.worldclim.org to construct the input dataset. The WorldClim parameters were chosen for combination because CHELSA and Soiltemp have some compatibility issues in Maxent models. For the Macroclimate model and projections, data were gathered from the CHELSA dataset, which is a ready-to-use model output of estimates of ERA-Interim climatic reanalysis downscaled with the CHELSA algorithm and is available at https://CHELSAclimate.org (Karger *et al*., 2022) with a 30 arcsec, (~1 km) resolution. Climatic variables for the 1981–2010 period were used to build the base Macroclimate model for the present. In addition to featuring measurements for a more recent period, the CHELSA dataset includes additional parameters that are biologically more relevant to *Ixodes* ticks, such as growth degree days above 5°C (Gdd5) (Jaenson and Lindgren, 2011; Gray *et al*., 2021), net primary productivity (Npp), which is related to vegetation cover important for ticks, growth season temperature (Gst) and growth season precipitation (Gsp). Additionally, since bioclimatic factors 8, 9, 18 and 19 were found to include some inconsistencies related to spatial artefacts first found in the WorldClim dataset and present in the CHELSA dataset (Escobar, 2020; Aguilar-Domínguez *et al*., 2021; Alkishe and Peterson, 2022), these variables were not used in datasets (Soiltemp, WorldClim and CHELSA). As the Mixed model dataset also included some macroclimatic variables (precipitation variables), the term ‘Mixed’ was chosen for differentiation from the other dataset, which included only macroclimatic variables. For the two datasets, variables were clipped to the area of interest between latitudes −20° and 65° and longitudes 20° and 65° WGS84. Geographic computations were performed with QGIS (QGIS Geographic Information System, 2022), the GDAL library in Python (Open Source Geospatial Foundation, 2022), and R version 4.2.2 (R Core Team, 2022).

### Future projections

As future projections are not available for microclimatic variables, GCMs available for the CHELSA dataset for three periods were used to construct future projections with the Macroclimate model selected for the CHELSA dataset: present-near future (2011–2040), near future (2041–2070) and distant future (2071–2100). The scenarios included SSP3-7.0 and SSP5-8.5 projections of UKESM1–0-LL (Sellar *et al*., 2019), MRI-ESM2-0 (Oshima *et al*., 2020), MPI-ESM1-2 (Mauritsen *et al*., 2019), IPSL-CM6-LR (Boucher *et al*., 2020) and GFDL-ESM4 (Dunne *et al*., 2020). Shared socioeconomic pathway (SSP) scenarios improve representative concentration pathways (RCPs) by including important socioeconomic parameters, such as urbanization, population growth and climate change (Hausfather, 2020). These datasets are also available at https://CHELSA-climate.org.

### Ecological niche models

Locations falling outside environmental rasters were removed before building the models. To reduce spatial sampling bias and to remove any unnoticed duplicates, the locations of *I. ricinus* were thinned to 10, 20, 30, 40 and 50 km, and five different location sets were obtained *via* the spThin package for R (Aiello-Lammens *et al*., 2015). The resulting datasets were split into a training set (70%), a cross-validation set for evaluating the candidate models (25%), and an independent test set for evaluating the final models (5%), which is crucial for revealing the real predictive power of a model. To better predict the distribution of a species, an accessible M space is needed (Soberon and Peterson, 2005; Barve *et al*., 2011). To construct the M space, a minimum convex polygon of a 100 km buffer area around the location points was created for the models using the ellipsenm package for R, which is available at https://github.com/marlonecobos/ellipsenm (Cobos *et al*., 2019), and environmental rasters were cut to this buffer area. For the Mixed model and the Macroclimate model, three sets were created for each of them by setting 0.9, 0.8 and 0.7 intervariable correlation thresholds using the vifcor function in the usdm package for R (Naimi, 2017), which eliminates variables from correlated pairs depending on the variance inflation factor (VIF), and by consulting the evaluations of the jackknife results of the preliminary Maxent models. An additional dataset was prepared for the Macroclimate model, depending on the previous models and jackknife results, for a total of three variable sets for the Mixed model and four sets for the Macroclimate model (Table 1).

**Table 1.** The environmental predictors used in different sets in the microclimate model and macroclimate model are explained in the first column

	Mixed				Macroclimate			
	Set 1	Set 2	Set 3		Set 1	Set 2	Set 3	Set 4
**Bio3**^a^ (isothermality)	√	x	x	**Bio3**^b^ (isothermality)	√	√	√	√
**Bio4**^a^ (temperature seasonality)	√	√	√	**Bio4**^b^ (temperature seasonality)	√	√	x	x
**Bio6**^a^ (minimum temperature of the coldest month)	√	√	√	**Bio5**^b^ (maximum temp. of the warmest month)	√	x	x	x
**Bio7**^a^ (annual range of air temperature)	√	x	x	**Bio6**^b^ (minimum temperature of the coldest month)	√	√	x	x
**Bio13**^c^ (precipitation amount of the wettest month)	√	√	√	**Bio7**^b^ (annual range of air temperature)	√	√	√	√
**Bio14**^c^ (precipitation amount of the driest month)	√	√	x	**Bio13**^b^ (precipitation amount of the wettest month)	√	√	√	√
**Bio15**^c^ (precipitation seasonality)	√	√	√	**Bio14**^b^ (precipitation amount of the driest month)	√	√	√	√
**Srad**^c^ (Solar radiation)	√	x	x	**Bio15**^b^ (precipitation seasonality)	√	√	√	√
**Vapr**^c^ (water vapour pressure)	√	√	√	**Gdd5**^b^ (growing degree days heat sum above 5°C)	√	√	x	x
				**Gst**^b^ (mean temperature of the growing season)	√	√	√	√
				**Gsp**^b^ (precipitation amount of the growing season)	√	√	√	√
				**Npp**^b^ (net primary productivity)	√	√	√	x

Data Sources.

a Soiltemp.

b CHELSA.

c WorldClim.

ENMs were built according to the maximum entropy algorithm (Phillips *et al*., 2006) and with the Kuenm R package (Cobos *et al*., 2019), which includes Maxent version 3.4.4 (Steven *et al*., 2021). The first calibration models were built with combinations of linear, quadratic, product and hinge features (L, Q, P, H) with regularization parameters of 0.1, 0.2, 0.3, 0.4, 0.5, 0.6, 0.7, 0.8, 0.9, 1, 2, 3, 4 and 5. To evaluate the models, a minimum threshold of 0.7 was set for the area under the curve (AUC), a maximum threshold of 0.05 was set for the omission rate, and a partial receiver operating characteristic (pROC) test was used (Peterson and Soberón, 2012; Aguilar-Domínguez *et al*., 2021). Further postanalysis model selection was carried out by the Akaike information criterion corrected for small sample sizes (AICc), and models with ΔAICc values less than 2 were selected as the final models (Warren and Seifert, 2011; Nuñez-Penichet *et al*., 2021). These final models were used to construct the final projections with 10 bootstrap replicates, and they were also tested with the independent testing locations that were set aside before model training and cross-validation.

Raster plots, which show the suitability of the distributions, were binarized using the 10th percentile training presence logistic threshold, which was taken from the Maxent results (Liu *et al*., 2005; Kramer-Schadt *et al*., 2013). For the future projections, 6 consensus maps were calculated by taking the median of the GCM scenarios for SSP3-7.0 and SSP5-8.5 for the periods 2011–2040, 2041–2070 and 2071–2100, respectively. To minimize possible deviations due to the novelty of the conditions compared with the original distribution area, the models were transferred to future conditions without any projection under extreme novel conditions (no extrapolation in the GCM scenarios) (Cobos *et al*., 2019). For the Mixed and Macroclimate models, the range (max val-min val) of the suitability maps of 10 bootstrap replications was used to create uncertainty maps. To map the uncertainty for future projections, we calculated the range of suitabilities between the different GCMs. Additionally, to determine the places where conditions are more extreme than in the calibration area of the models, a mobility-oriented parity (MOP) analysis was carried out, and the extrapolation risk of transfer regions was calculated with the nearest 10% reference *via* the MOP package for R (Owens *et al*., 2013; Alkishe *et al*., 2020; Flores-López *et al*., 2022). Three types of MOP maps were created, (1) representing how many variables in the region of projection are non-analogous to those in the calibration area and where non-analogous conditions were found towards (2) high values and (3) low values of each variable were created for each GCM. All the maps were created with QGIS software and R software.

## Results

### Model parameters

**Figure 1.**
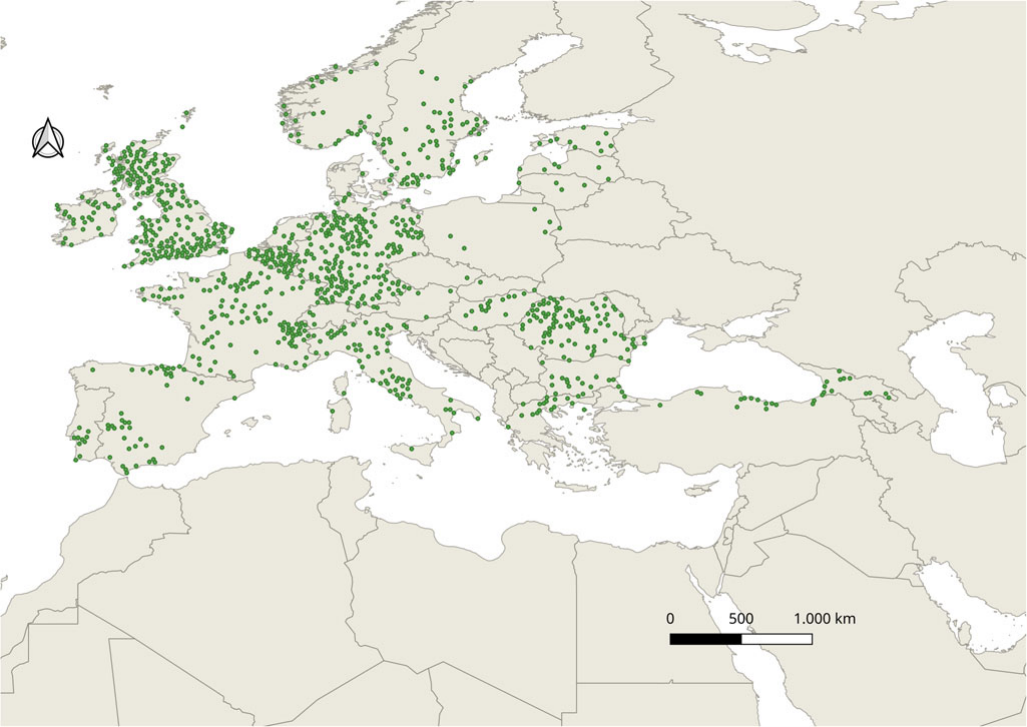
All occurrence points of *Ixodes ricinus* after cleaning and thinning.

As models that used locations thinned to a greater distance than 20 km had AUC values less than 0.7, models created with 20 km thinned locations were selected and had a total of *n* = 1001 locations (Fig. 1). Among these locations, ~70% (*n* = 713) were used to train the models, ~25% (*n* = 238) were used for cross-validation, and ~5% (*n* = 50) were set aside for the final evaluation tests. For the Mixed model, out of all 630 models, 627 were statistically significant, while only 24 passed the 0.05 omission rate criterion, and only one model from these fulfilled the AICc. The final model was a linear product (LP) model with a regularization multiplier of 0.5; this model used the second set, with intercorrelations less than 0.8 and had an omission rate of 0.046, a pROC value of ~0 and an AUC ratio of 1.16. The final evaluations performed on the independent test set showed an omission rate of 0.06, which is marginally above 0.05; on the other hand, this means that the Mixed model correctly classified approximately 47 points out of 50 points from the independent test locations. The contributions of the 6 environmental variables to the final Mixed model are shown in Table 2. The most important environmental variable was Bio4, the temperature seasonality near the soil, with a contribution of 40.6%, followed by vapour pressure, which contributed 36%, and Bio14 (precipitation amount in the wettest month) and Bio15 (precipitation seasonality), both of which contributed 9.6%. Bio6, the near-soil minimum temperature of the coldest month, had a 3.3% contribution.

**Table 2.** Percent contribution of the environmental predictors to the models

Mixed		Macroclimate	
Predictor	Percent contribution	Predictor	Percent contribution
Bio4^a^	40.6	Bio7^b^	52
Vapr^c^	36	Gsp^b^	26.5
Bio14^c^	9.6	Bio15^b^	12.4
Bio15^c^	9.6	Bio3^b^	5.6
Bio6^a^	3.3	Gst^b^	1.4
Bio13^c^	0.9	Bio13^b^	1.1
		Bio14^b^	1

Data Sources.

a Soiltemp.

b CHELSA.

c WorldClim.

For the Macroclimate dataset, the AUC also fell below 0.7 beyond a 20 km thinning length in the models generated using the CHELSA 1981–2010 dataset; thus, the 20 km thinned locations were also used in the models. Out of the 840 candidate models, 757 were statistically significant, 49 passed the 0.05 omission rate criterion, and only one fulfilled both the omission rate and AICc conditions. The final Macroclimate model was a linear quadratic product hinge (LQPH) model combining all features with a regularization multiplier of 2 that used the 4th variable set (Table 2), which had a 0.7 upper intercorrelation threshold. The model had an omission rate of 0.048 and a pROC of ~0, with an AUC of 1.14. Final evaluations with the independent test revealed that the Macroclimate model had better results in predicting the independent test results, with a final evaluation omission rate of 0.041, which is even better than the cross-validation omission rate of the same model. Although this result is better than that of the final evaluations of the Mixed model, the values are actually very close, as the 0.041 omission rate means that it correctly assigned 48 out of the 50 independent test locations correctly. According to the Macroclimate model, the largest contributor was Bio7 (annual range of air temperature), which contributed 52% of the total contribution. This was followed by Gsp (growth season precipitation), which contributed 26.5%, whereas Bio3 (isothermality) contributed 5.6%.

### Current and future predictions

**Figure 2.**
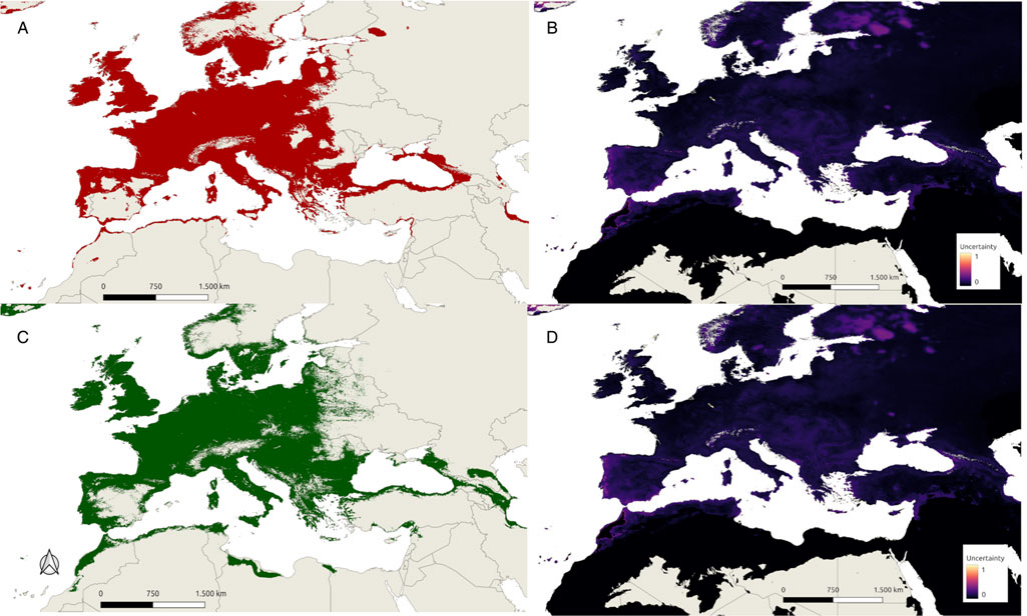
Maps of predicted suitable areas for *I. ricinus* and uncertainty from the ENM results. (A) Red areas show the suitable regions under current conditions according to the Macroclimate model. (B) Uncertainty values of the Macroclimate model. (C) Green areas show the suitable regions under current conditions according to the Mixed model. (D) Uncertainty values of the Mixed model.

For the current prediction maps, the binary thresholds were 0.27 and 0.28, respectively, with very similar 10^th^ percentile training presence logistic threshold values for both the Mixed and Macroclimate models. The predicted areas of suitability and uncertainty are shown in Fig. 2. Both the Mixed and Macroclimate models predicted a wide distributional area that covers much of Europe except for gaps in mountainous areas and parts of southern Europe, especially Spain and Greece. The climatically suitable regions mostly coincided with the current distribution of *I. ricinus*. On the other hand, in western Asia, suitable regions are located on the coast of the Black Sea. The Mixed model revealed a wider suitable region in the Caucasus, whereas the Macroclimate model for 1981–2010 predicted a smaller suitable region in the southern Caspian Sea. Both models also predicted a small suitable region on the coast of North Africa, with the Mixed model showing a slightly larger area in North Africa. On the other hand, the level of uncertainty is much greater in southern Europe and North Africa.

**Figure 3.**
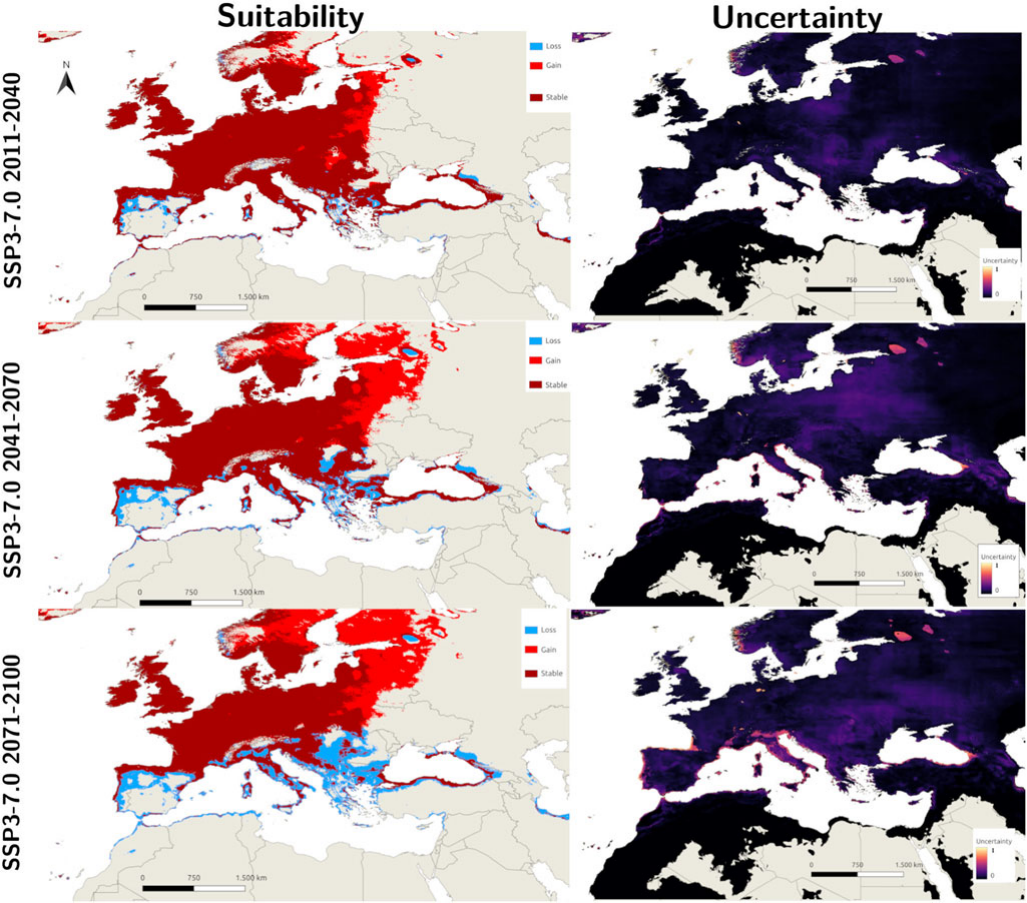
Side-by-side predicted suitable areas for *I. ricinus* and uncertainty values for the median of 5 GCM scenarios for SSP3-7.0 with differing degrees of loss and gain compared with the current conditions (1981–2010) for the Macroclimate model.

**Figure 4.**
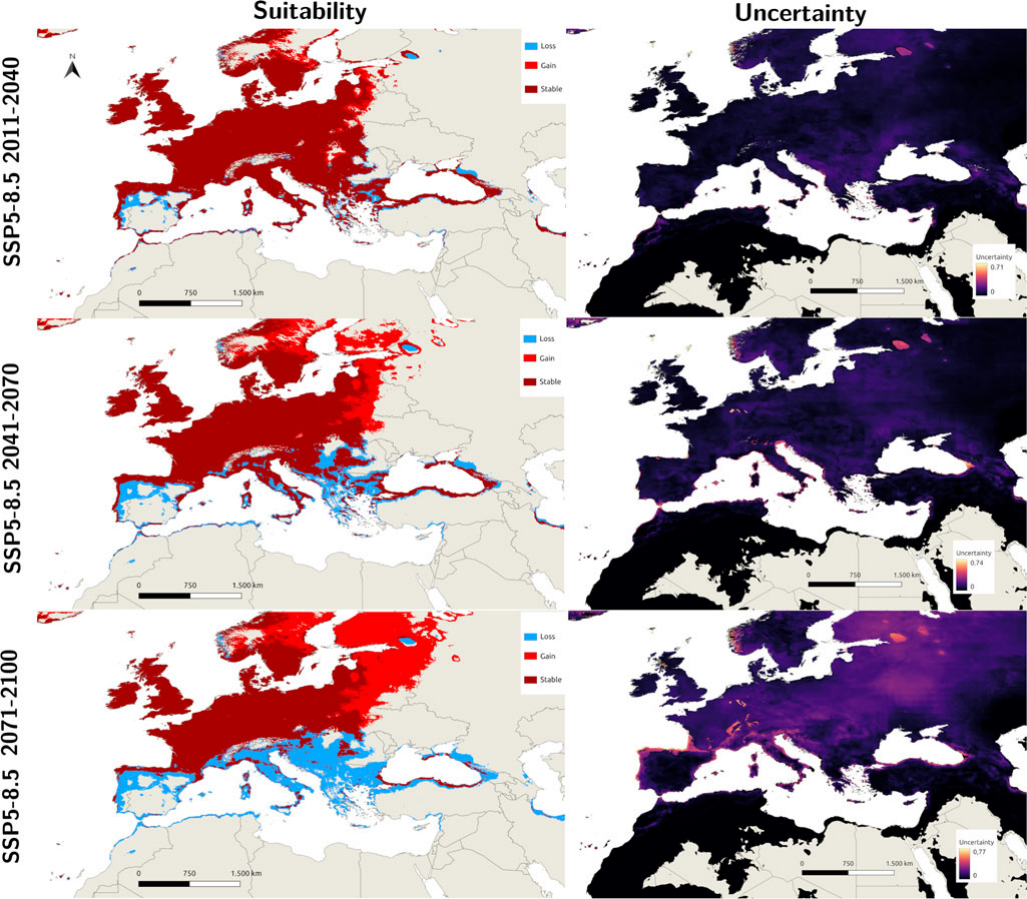
Side-by-side predicted suitable areas for *I. ricinus* and uncertainty values for the median of 5 GCM scenarios for SSP5-8.5 with differing degrees of loss and gain compared with the current conditions (1981–2010) for the Macroclimate model.

The consensuses of the different GCM future projections are shown in Figs 3 and 4, and each individual future projection is included in Supplementary File 1. The predictions of SSP3-7.0 for the 2011–2040 period reveal significant expansion in eastern and northern Europe, whereas there are some declines in southern Europe, the Balkans and the Black Sea region. Interestingly, despite being a more extreme scenario, the SSP5-8.5 projection for 2011–2040 showed a smaller area of expansion, while the pattern of decline was similar to that of the SSP3-7.0 scenario. The level of uncertainty increases from western Europe to the peripheral regions where most of the expansion and decline occurs. Future projections for 2041–2070 suggest the continuation of increasing suitability in northeastern Europe, with the SSP3-7.0 scenario showing a spread that is slightly greater than that of SSP5-8.5. Conversely, the decreases in the southern region are greater in the SSP5-8.5 projection for 2041–2070. Distant future projections for 2071–2100 show that this trend of decreased suitability in south and increase in the north is expected to continue in both scenarios, with the SSP5-8.5 projection depicting a very extreme decline in the south, where most of the Mediterranean and Balkans will not be suitable for *I. ricinus.*

### Uncertainty and MOP results

For the present conditions, the uncertainty of the mixed model is relatively low within the main distributional area of *I. ricinus*, whereas the highest levels of uncertainty are observed at the margins (Fig. 2), particularly in the eastern Mediterranean, southern Caucasus (Georgia), North African Coast, southwestern Spain and western Scandinavia. Uncertainty attains the highest values in the eastern Mediterranean and North Africa, outside the distribution of *I. ricinus*. Therefore, the high suitability results in these areas are not very reliable. For the Macroclimate model, although the maximum level of uncertainty is greater than that of the Mixed model, the overall level of uncertainty is lower, as shown in Fig. 2. The general pattern of uncertainty is similar and increases towards the margins. Specifically, east of the Mediterranean, North Africa, west of Spain and western Scandinavia present higher levels of uncertainty. Additionally, the uncertainty in future projections is greater than that in present projections. However, the pattern of uncertainty remains similar to that at present, with western Europe (including France, the United Kingdom and parts of Germany) experiencing the lowest uncertainty levels, whereas higher uncertainty is evident around the Mediterranean basin, the Balkans, western Spain and western Scandinavia. The general level of uncertainty is very high in the 2071–2100 projections, particularly in the SSP5-8.5 projections; additionally, there are more areas with extreme uncertainty, suggesting that distant future projections (especially the worst-case scenarios) are not very reliable for tick distribution models (see Figs 3 and 4).

The MOP results closely mirror other uncertainties and are included in Supplementary File 2. In the 2011–2040 and 2041–2070 SSP3-7.0 and SSP5-8.5 future projections, areas with significant extreme extrapolation begin at the south of Anatolia to Iran and the Middle East, North Africa and east of the Caspian Sea. Almost all regions with significant extrapolations fall outside the current and predicted future distribution range of *I. ricinus*, except for small areas in southern Anatolia and North Africa. For the 2071–2100 projections, in accordance with the uncertainty maps, the areas with extreme extrapolation are also dispersed to the coast of Mediterranean regions, including Spain, Italy, Greece and Turkey, which is more prominent in the SSP5-8.5 projection.

## Discussion

*Ixodes ricinus* has high adaptability to European environments and a tendency to expand its geographic range towards northern altitudes, which is mostly explained by climate change (Materna *et al*., 2008; Jaenson and Lindgren, 2011; Martello *et al*., 2014; Garcia-Vozmediano *et al*., 2020; Hvidsten *et al*., 2020). This raises the question of whether this expansion is likely to give rise to new, distant foci for Lyme and TBE diseases. Environmental parameters play a significant role in influencing the distribution of *I. ricinus*. Factors such as climatic conditions, habitat characteristics, and landscape heterogeneity have been identified as key determinants affecting the abundance and prevalence of this tick species (Krasnov *et al*., 2007; Ruiz-Fons *et al*., 2012; Hauser *et al*., 2018).

The Mixed model identified temperature seasonality as the primary environmental predictor, whereas the Macroclimate model indicated that the annual temperature range was the most significant contributor. These findings are consistent with those of previous studies. The annual temperature range, annual mean temperature and annual precipitation were identified as the most influential factors by Alkishe *et al*. (2017). Similarly, temperature seasonality is considered a relevant factor according to Cunze *et al*. (2022). The second most influential factor is vapour pressure and growth season precipitation according to the Mixed and Macroclimate models, respectively. The off-host survival of ticks depends strongly on water availability since desiccation is one of the most prominent causes of tick mortality and decreasing questing activity (Perret *et al*., 2004; Tagliapietra *et al*., 2011; Nolzen *et al*., 2022; Van Gestel *et al*., 2022). Factors such as higher soil moisture content and increased cloud cover have been linked to increased questing activity in *I. ricinus* (Medlock *et al*., 2008; Lauterbach *et al*., 2013). Additionally, the distribution of *I. ricinus* has expanded in the past three decades due to more favourable biotic and abiotic conditions, which can be influenced by changes in precipitation and an increase in mean winter temperatures (Gray and Ogden, 2021). Other modelling studies have underscored the importance of precipitation as a significant factor in the distribution of this species in both current and future projections (Alkishe *et al*., 2017; Cunze *et al*., 2022). Temperature has been recognized in various studies as a crucial determinant of the completion of the life cycle and duration of the host-seeking activity of *I. ricinus* at northern latitudes (Lindgren *et al*., 2000; Schwarz *et al*., 2012; Hauser *et al*., 2018). However, Gst (growth season temperature) made a very small contribution to the final Macroclimate models, and Gdd5 (degree days above 5°C) was not included in the final selected models. Another discrepancy with these previous studies is that the minimum temperature of the coldest month (Bio6) contributed less than other variables in the Mixed model; moreover, in the final selected models for Macroclimate, Bio6 was not included because of correlation thresholds. However, when Bio6 was included in the side models, Bio6 was again behind other parameters, with a contribution smaller than 3%. This might be due to the difference in the CHELSA and Soiltemp datasets used in creating the models. Additionally, the relationship between minimum temperature and tick survival might be more complicated because of the buffering effect of microclimatic factors such as snow cover and leaf litter on tick survival and activity (Van Gestel *et al*., 2022). For example, *in situ* measurements have shown that the humidity and temperature in the understorey of forests where *I. ricinus* resides are indeed much more mild and stable than those measured from standard weather stations (Boehnke *et al*., 2015). Additionally, the increased winter mortality effect of removing leaf litter and snow cover on *Ixodes scapularis* was documented in experimental field plots (Volk *et al*., 2022).

It might be expected that microclimatic parameters are more accurate for predicting the distribution of ticks because of the proven strong connection of tick survival with microclimatic abiotic factors in the environment; however, the omission rates of the distribution models are very similar to those of the Macroclimate model, which has a very slight edge. This could be attributed to several factors. One explanation might be that the two microclimatic parameters that were included in the final mixed model may not be good predictors; however, on the contrary, Bio4 was the greatest contributor to the model, which supports the predictive power of macroclimatic parameters. Notably, the Mixed model also had very good predictive results. A more plausible explanation might be that the Macroclimate model included additional important parameters that improved the power of the model. Gsp was the second most important contributor to the Macroclimate model, which is not surprising given that most tick species are active in the growing season. In addition, the second most important parameter for the Mixed model was water vapour pressure from WorldClim, a macroclimatic dataset. The Soiltemp dataset includes only temperature-related variables, and precipitation is the same for macro- and microclimate. Thus, the inclusion of additional parameters related to water content and humidity (crucial factors for ticks) at the microclimate level would improve these models since relative humidity often varies with microclimate, at least as much as temperature (Van Gestel *et al*., 2022).

The models indicate that the region climatically suitable for *I. ricinus* covers a large portion of Europe except southern Spain and Greece and the coastal regions of the Black Sea in West Asia. This outcome is consistent with the results of previous models of *I. ricinus*. On the other hand, model predictions revealed a more constrained distribution in the southern regions of Europe, especially in southern and western Anatolia; this is an expected result, as we know that there is presently no record of *I. ricinus* in this region (Hekimoğlu, 2022). The model also revealed a small area of suitability on the North African coast; however, the suitability of this region is not reliable due to the high degree of uncertainty. Although some of the previous studies included *I. ricinus* from North Africa, there is a high probability that some of these records involved *I. inopinatus,* as recent genomic analysis clearly demonstrated the presence of *I. inopinatus* in North Africa (Younsi *et al*., 2020; Rollins *et al*., 2023). Nevertheless, the absence of current and future distributions of *I. ricinus* in the Mediterranean region cannot be ascribed solely to *I. inopinatus*. Although the distribution of *I. inopinatus* in different European countries, such as Germany, Spain and Portugal, has been suggested (Estrada-Peña *et al*., 2014; Chitimia-Dobler *et al*., 2018; Hauck *et al*., 2019), genomic data have shown that German samples are in fact *I. ricinus* (Rollins *et al*., 2023). In this case, the taxonomic status of Mediterranean populations should be reevaluated, as this could influence the projections of *I. ricinus* in these areas. Considering the taxonomic reevaluation mentioned above, many recent studies excluded North African populations from their dataset (Cunze *et al*., 2022; Noll *et al*., 2023). The findings of these studies largely align with our current and future predictions. A large part of Europe constitutes the distribution area of the species, with the most suitable areas located in Western Europe. While Mediterranean countries such as France and Italy include suitable areas for the species, Portugal and the northern part of Spain also appear suitable in the western Mediterranean. Along the Black Sea coast, the most suitable areas include coastal regions encompassing the northern part of Turkey.

Future projections indicate a spread in northern and eastern Europe, confirming other projections with different bioclimatic datasets (Cunze *et al*., 2022; Noll *et al*., 2023). A consensus is also reached with areas that will not be suitable in the future, particularly for the predicted habitat loss in Spain, Greece and the Balkans (Cunze *et al*., 2022). Another outcome is that in the present–near-future scenarios (2011–2040), the midway scenario (SSP3-7.0) estimates a slightly wider new distributional area for *I. ricinus* in northeastern Europe than does SSP5-8.5, a worse-case scenario. This outcome is compatible with the previous future projections by Porretta *et al*. (2013) and also Alkishe *et al*. (2017), where midway scenarios also predicted a wider new region of suitability in the near future. While the spread in the 2041–2070 scenarios is similar between the SSP3-7.0 and SSP5-8.5 scenarios, the decline in the southern distributional areas, including the Balkans and Mediterranean regions, is much more prominent in SSP5-8.5 than in the other regions, which is most likely due to the predictions of great declines in precipitation in this region. Distant future scenarios (2071–2100) continue this increase in suitability towards the north, whereas the decline in southern regions becomes much more excessive (especially in the SSP5-8.5 scenario). On the other hand, the level of uncertainty and the degree of extrapolation are also extreme in these regions for this period, and thus, it would be inaccurate to reach any assumption for this period. Several climatic studies in recent decades since the early 2000s have deemed the Mediterranean basin a climate change hotspot, with projections showing increased temperature and aridity, heightened vulnerability to drought and high temperatures and a greater frequency of heat waves (Ulbrich *et al*., 2006; Giorgi and Lionello, 2008; Naumann *et al*., 2018). Furthermore, the impacts of recent climate change on increased drought frequency and magnitude have already been documented in Mediterranean-type climates (Hoerling *et al*., 2012; Feng *et al*., 2019). Current projections indicate that the most suitable distribution areas for the species in Turkey are the coasts of the Black Sea and the Thrace region. However, while smaller suitable areas are also indicated along the Mediterranean coast of the country, the uncertainty in these predicted areas is again quite high. Future projections suggest a loss of suitability on the southern coast and, additionally, decline in certain localities, especially in the inland areas of Thrace in the north. This pattern indicates that expected climate changes in the Mediterranean Basin in the future will result in a decrease in the distribution areas of this species in Turkey, similar to the projections for Spain.

Ecological niche models are valuable tools for assessing the possible presence and future distribution of parasite vectors; however, several caveats exist, and special care is needed when building distribution models. Major challenges are the potential bias and limitations in the outputs of niche models, especially when projecting to future scenarios (Peterson *et al*., 2018); therefore, providing the necessary uncertainty values associated with these models is crucial. Additionally, ectoparasites, including ticks, partially depend on their hosts for survival and transportation, posing other risks in modelling. Host species and their abundance are among the most important factors influencing the distribution of ticks to new areas and establishing populations. Moreover, the large number of migrating birds increases the probability of the geographic spread of *I. ricinus* and related diseases, emphasizing the importance of considering avian hosts in disease epidemiology (Waldenström *et al*., 2007; Ciebiera *et al*., 2019). Although hosts play a crucial role in the transmission of ticks, several limitations are associated with including hosts in modelling studies. For example, *I. ricinus* is a three-host species that feeds on different hosts during its life cycle (Hofmeester *et al*., 2016), which can introduce complexity when multiple hosts are used in modelling studies. Recent papers have presented contrasting hypotheses regarding tick–host associations. Some suggest that ticks select hosts on the basis of the environment, whereas others propose that ticks select environments and feed on any available host within those environments (Nava and Guglielmone, 2013; Zhang *et al*., 2019; Ginsberg *et al*., 2022; Estrada-Peña *et al*., 2023). Moreover, while it has been suggested that hosts can influence tick distribution at a smaller geographical scale, the distribution of ticks over a wide spatial scale is determined primarily by direct climatic effects rather than by host presence (Cumming, 2002). Considering all these factors, understanding the interactions between hosts, the environment and ticks is essential for developing effective strategies to manage tick-borne diseases and control their spread. On the other hand, a very recent study by Fabri *et al*. (2024) revealed that *I. ricinus* depends much more on abiotic factors than on the composition of its hosts.

## Conclusion

This study aimed to update previous projections by reviewing coordinates, utilizing new datasets and additionally implementing microclimatic parameters to construct assistive models that complement other distribution models that rely on macroclimatic parameters. According to the results, the Mixed model, combining micro and macro parameters achieved similar levels of accuracy with the Macroclimate model. However, the integration of microclimates in distribution models is still in its starting phase, and the inclusion of additional microclimatic parameters in available datasets, updating the presently available variables with new data and also widening these models to other species will be beneficial. For species distribution models, we still depend on macroclimatic datasets since predictions of very fine-grained parameters such as the microclimate in the future are not yet available, and macroclimate data still provide good predictions at broader scales. Therefore, it is essential to continuously replicate, validate and update previous models to better predict the possible future distributions of disease-transmitting ticks.

## Supporting information

Kuyucu and Hekimoglu supplementary material 1 Kuyucu and Hekimoglu supplementary material

Kuyucu and Hekimoglu supplementary material 2 Kuyucu and Hekimoglu supplementary material

## Data Availability

The coordinates used in this study are available at Mendeley Data. https://data.mendeley.com/datasets/98m45vcfp2/1

## Supplementary material

The supplementary material for this article can be found at https://doi.org/10.1017/S003118202400132X
