# Supplementary material for: Predicting the distribution of Ixodes ricinus in Europe: integrating microclimatic factors into ecological niche models
Source: Parasitology. 2024 Nov 7;151(9):1012–23. doi: 10.1017/S003118202400132X (PMC11770518; doi:10.1017/S003118202400132X)
Supplement: Kuyucu and Hekimoglu supplementary material 1 — Kuyucu and Hekimoglu supplementary material [file S003118202400132Xsup001.pdf]

2011-2040

GFDL-ESM4 SSP3-7.0

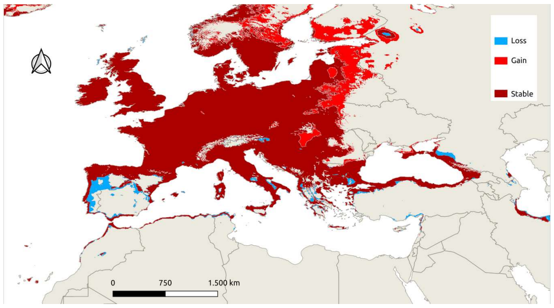

GFDL-ESM4 SSP5-8.5

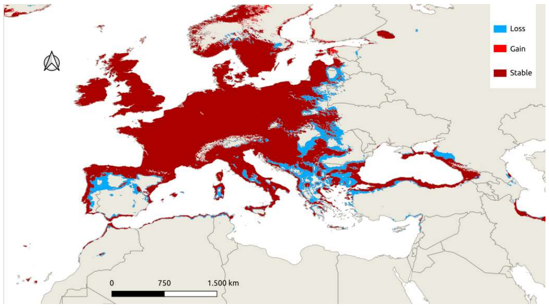

IPSL-CM6A-LR SSP3-7.0

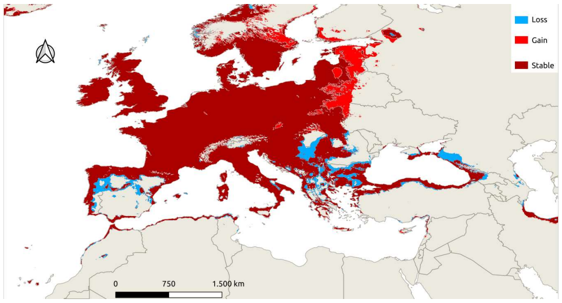

IPSL-CM6A-LR SSP5-8.5

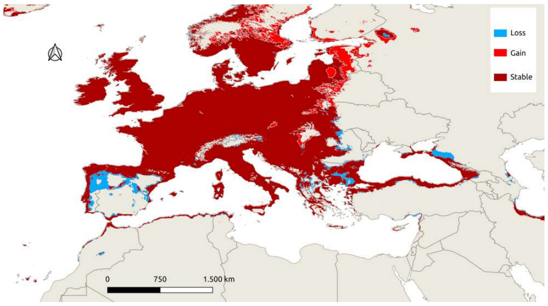

MPI-ESM1-2-HR SSP3-7.0

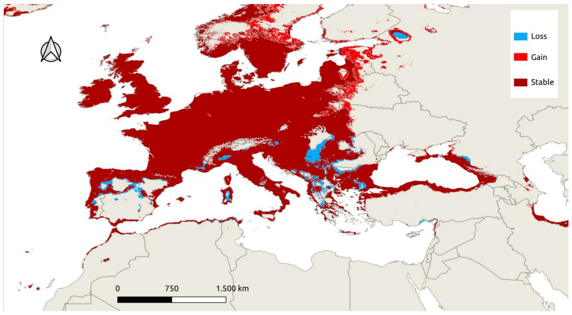

MPI-ESM1-2-HR SSP5-8.5

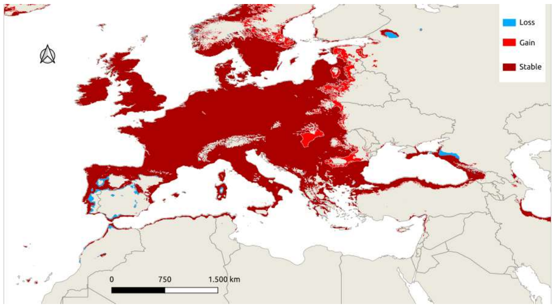

MRI-ESM2-0 SSP3-7.0

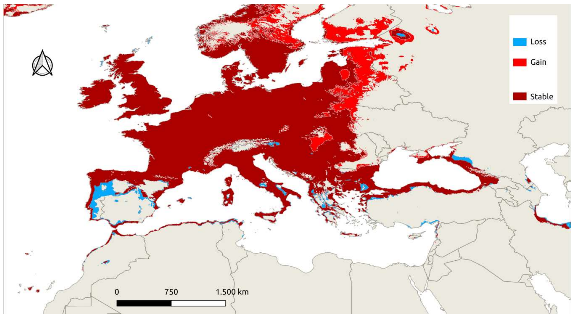

MRI-ESM2-0 SSP5-8.5

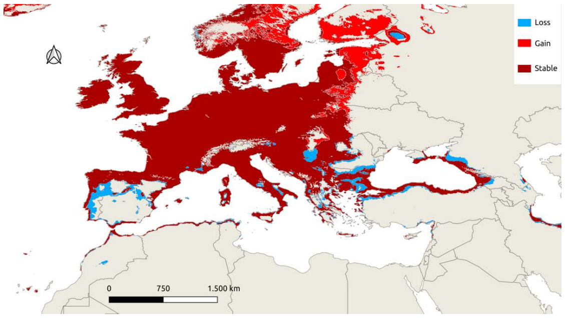

UKESM1-0-LL SSP3-7.0

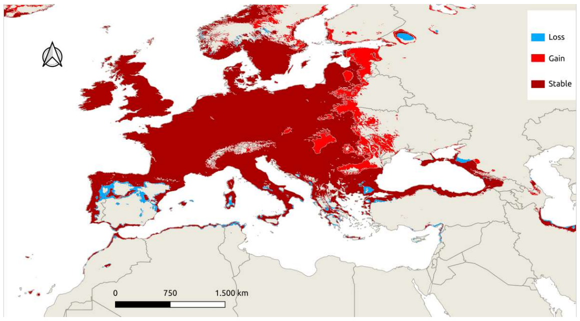

UKESM1-0-LL SSP5-8.5

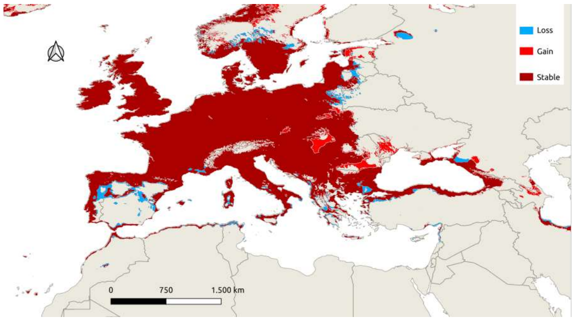

2041-2070

GFDL-ESM4 SSP3-7.0

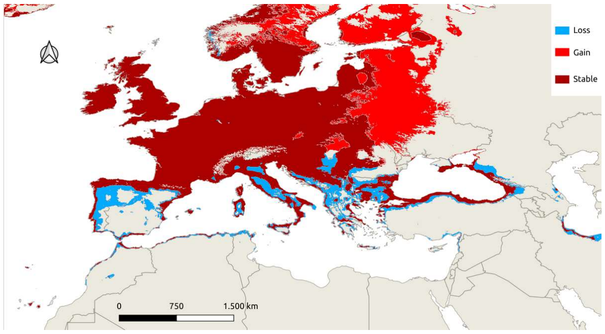

GFDL-ESM4 SSP5-8.5

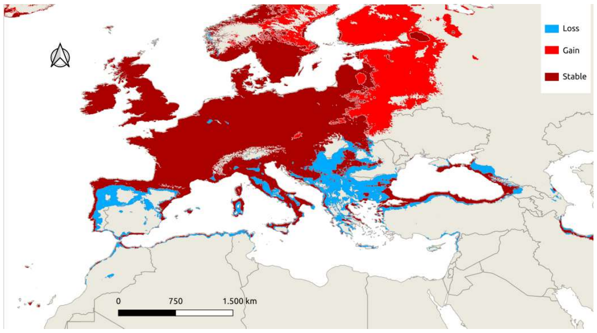

IPSL-CM6A-LR SSP3-7.0

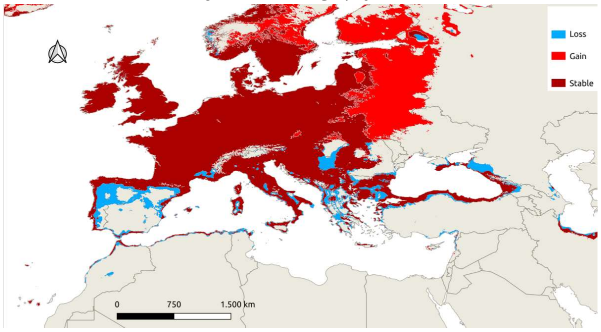

IPSL-CM6A-LR SSP5-8.5

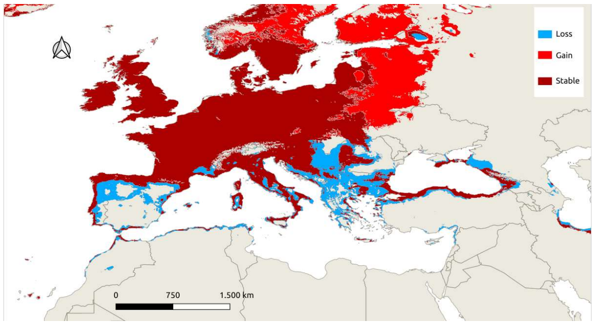

MPI-ESM1-2-HR SSP3-7.0

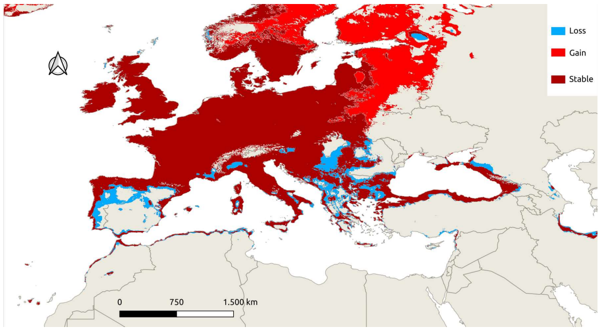

MPI-ESM1-2-HR SSP5-8.5

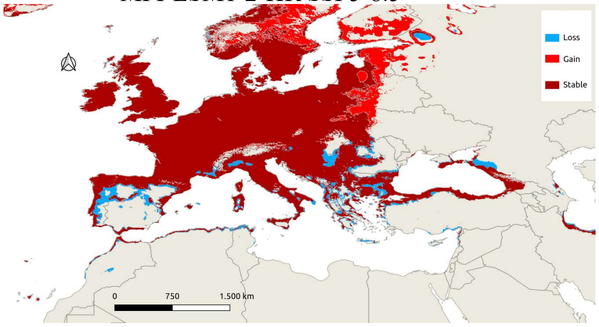

MRI-ESM2-0 SSP3-7.0

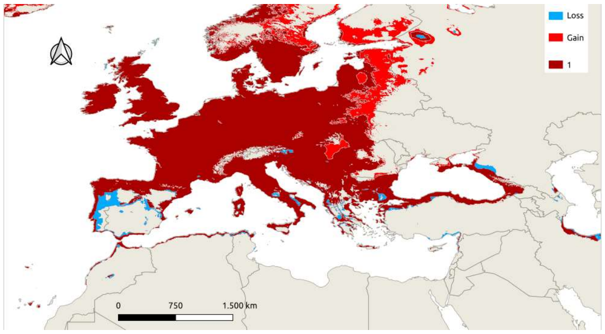

MRI-ESM2-0 SSP5-8.5

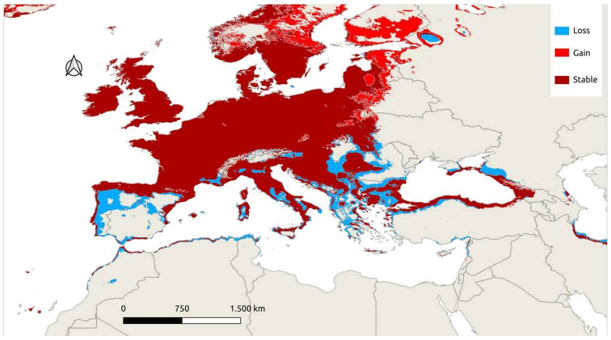

UKESM1-0-LL SSP3-7.0

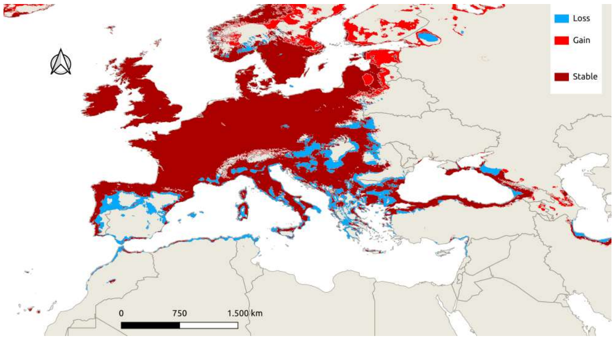

UKESM1-0-LL SSP5-8.5

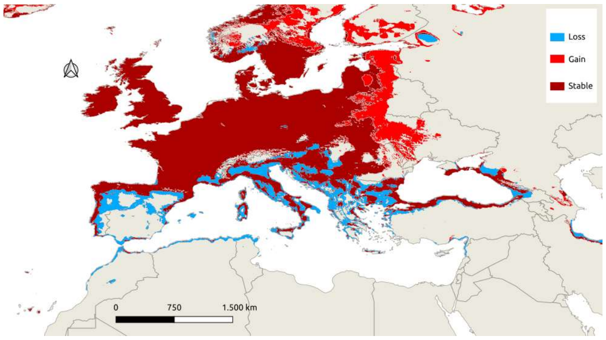

2071-2100

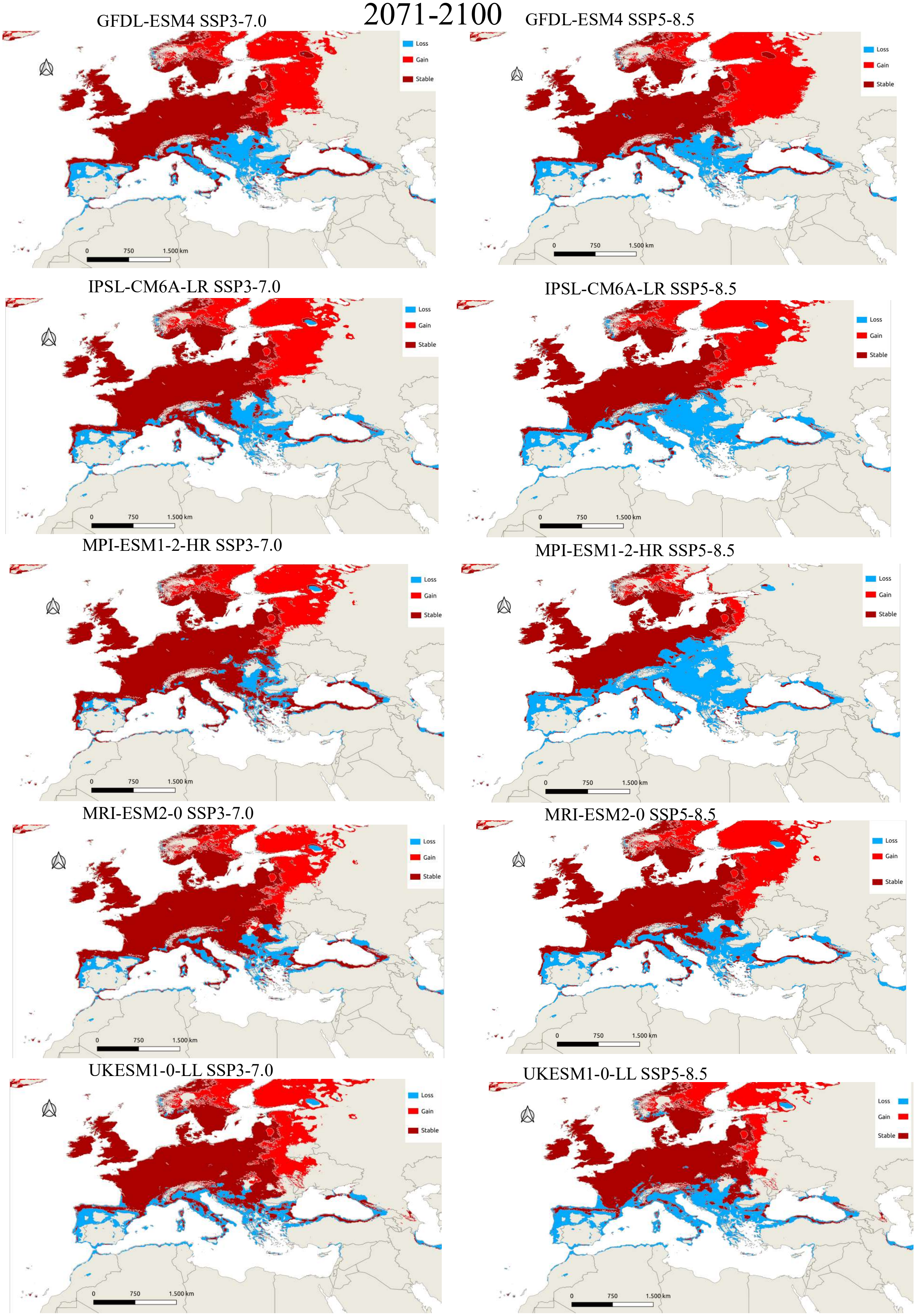
GFDL-ESM4 SSP3-7.0
GFDL-ESM4 SSP5-8.5
IPSL-CM6A-LR SSP3-7.0
IPSL-CM6A-LR SSP5-8.5
MPI-ESM1-2-HR SSP3-7.0
MPI-ESM1-2-HR SSP5-8.5
MRI-ESM2-0 SSP3-7.0
MRI-ESM2-0 SSP5-8.5
UKESM1-0-LL SSP3-7.0
UKESM1-0-LL SSP5-8.5
Loss
Gain
Stable
0
750
1.500 km
