# Supplementary material for: Predicting the distribution of Ixodes ricinus in Europe: integrating microclimatic factors into ecological niche models
Source: Parasitology. 2024 Nov 7;151(9):1012–23. doi: 10.1017/S003118202400132X (PMC11770518; doi:10.1017/S003118202400132X)

A) MOP for all variables representing how many variables in the set of interest are non-analogous to those in the reference set

2011-2040

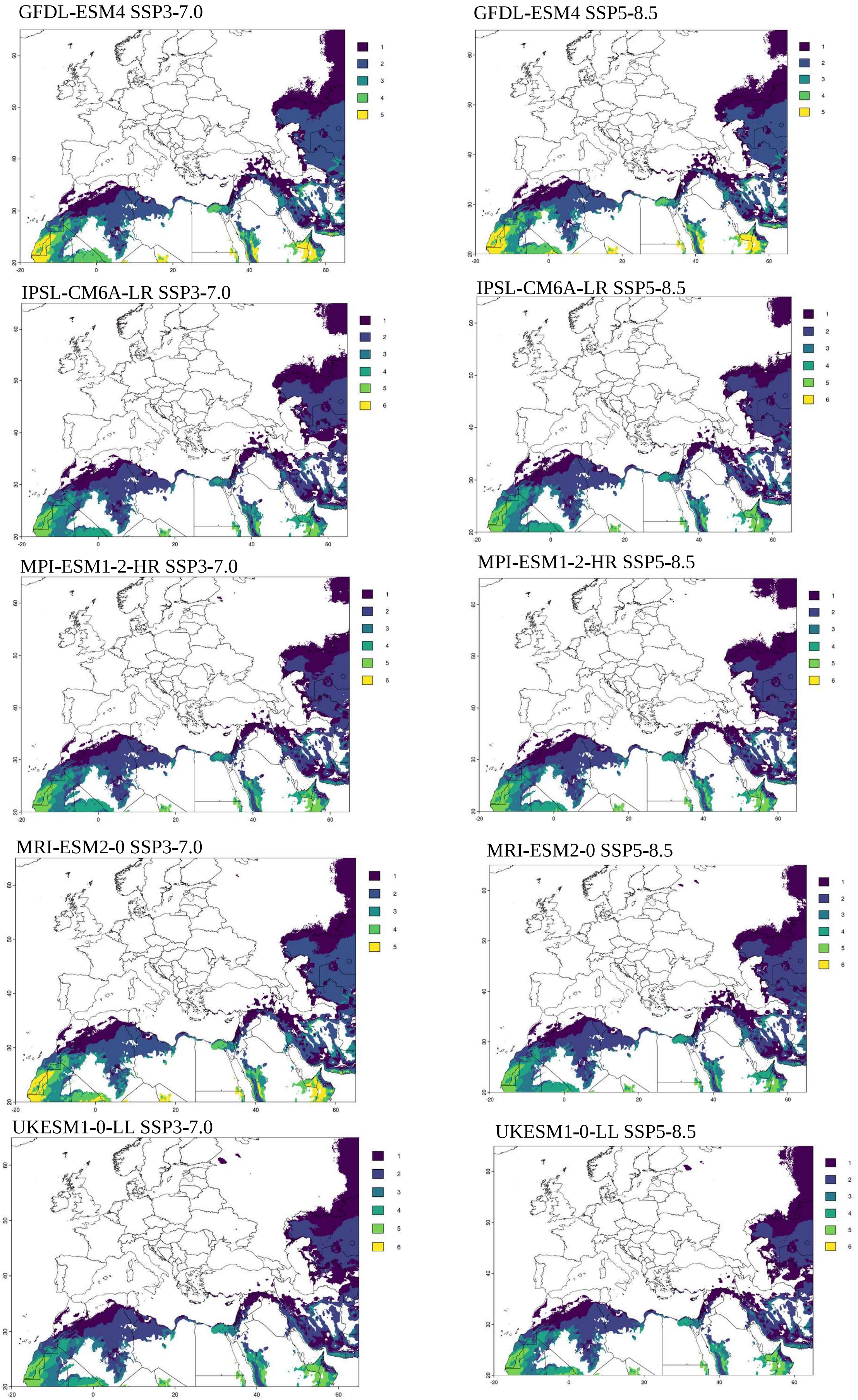

2041-2070

GFDL-ESM4 SSP3-7.0

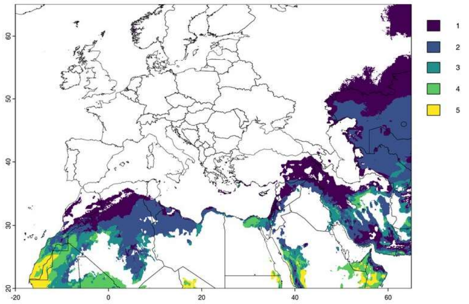

GFDL-ESM4 SSP5-8.5

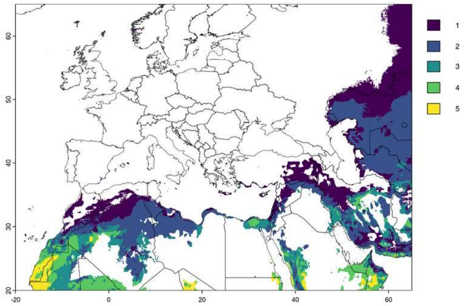

IPSL-CM6A-LR SSP3-7.0

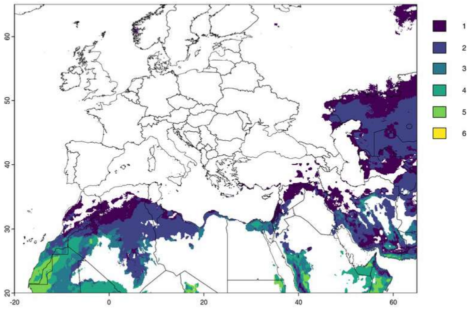

IPSL-CM6A-LR SSP5-8.5

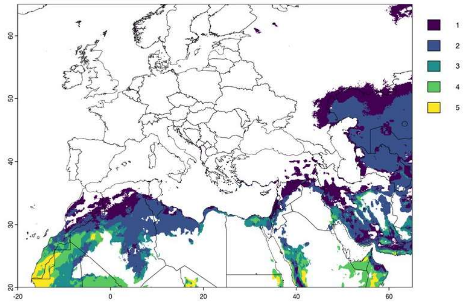

MPI-ESM1-2-HR SSP3-7.0

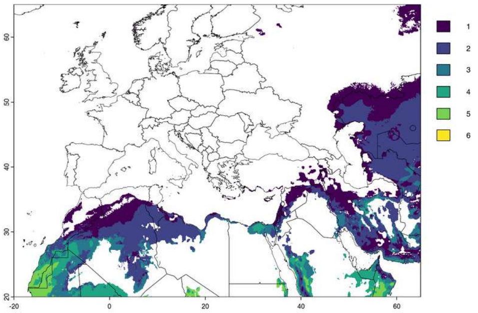

MPI-ESM1-2-HR SSP5-8.5

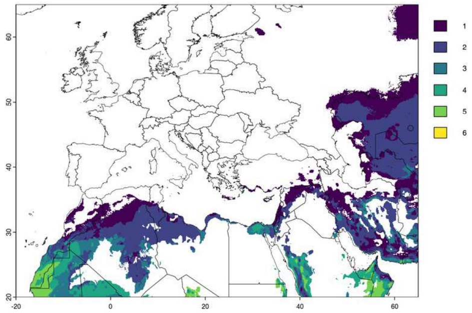

MRI-ESM2-0 SSP3-7.0

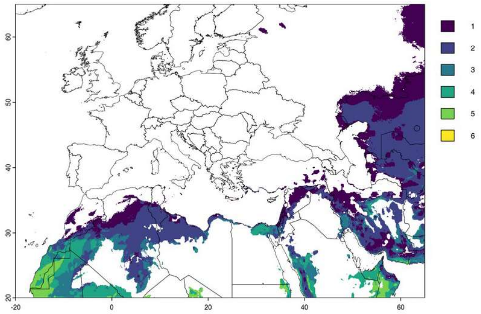

MRI-ESM2-0 SSP5-8.5

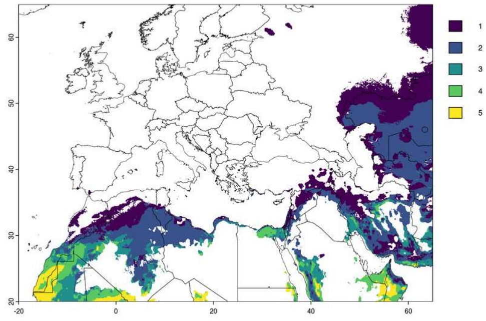

UKESM1-0-LL SSP3-7.0

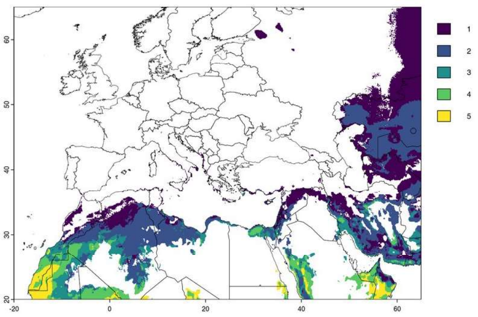

UKESM1-0-LL SSP5-8.5

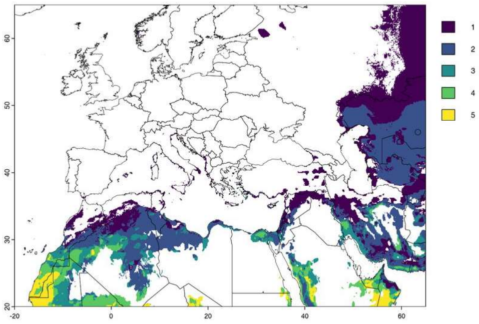

2071-2100

GFDL-ESM4 SSP3-7.0

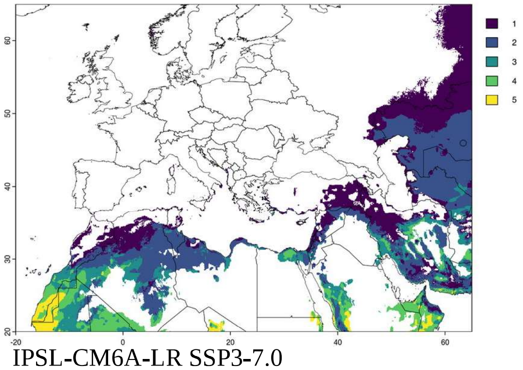

GFDL-ESM4 SSP5-8.5

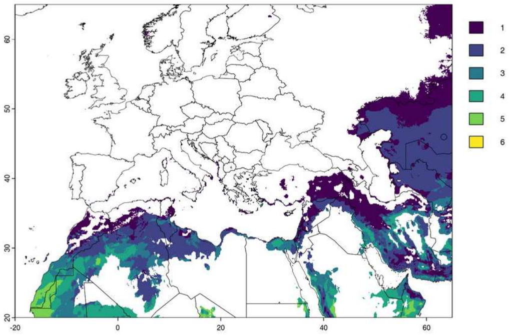

IPSL-CM6A-LR SSP3-7.0

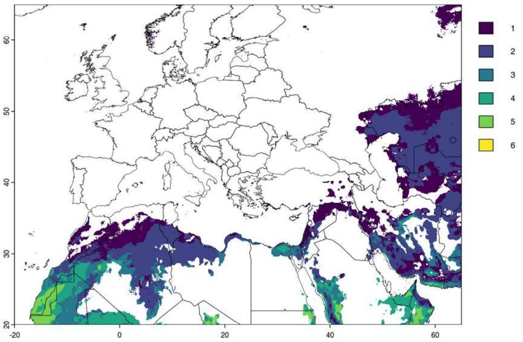

IPSL-CM6A-LR SSP5-8.5

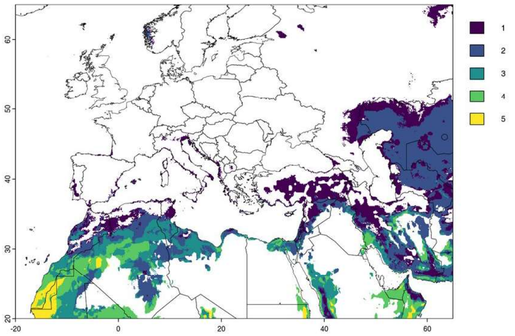

MPI-ESM1-2-HR SSP3-7.0

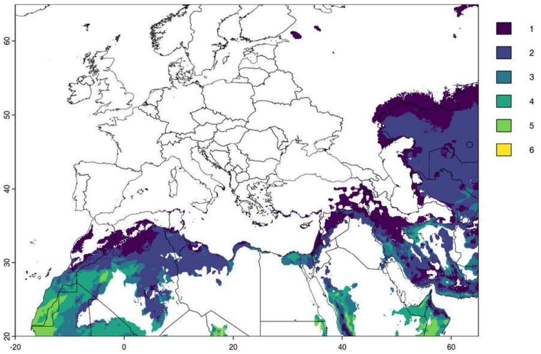

MPI-ESM1-2-HR SSP5-8.5

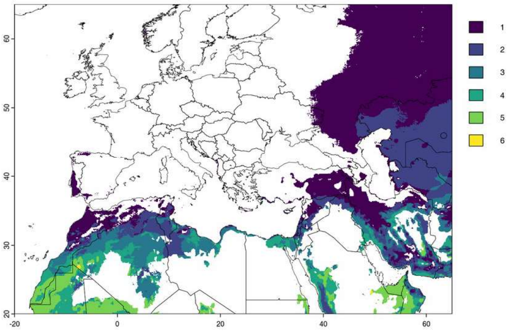

MRI-ESM2-0 SSP3-7.0

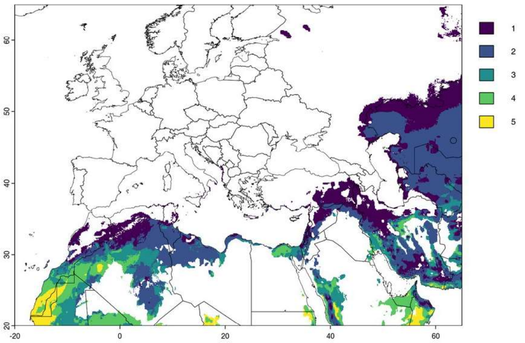

MRI-ESM2-0 SSP5-8.5

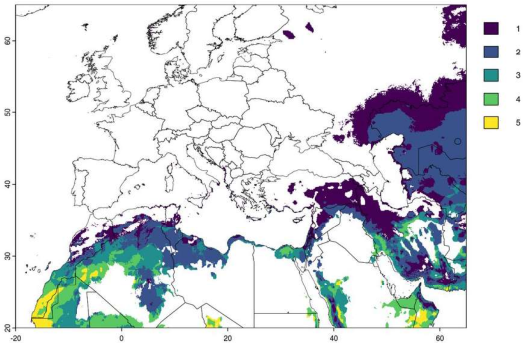

UKESM1-0-LL SSP3-7.0

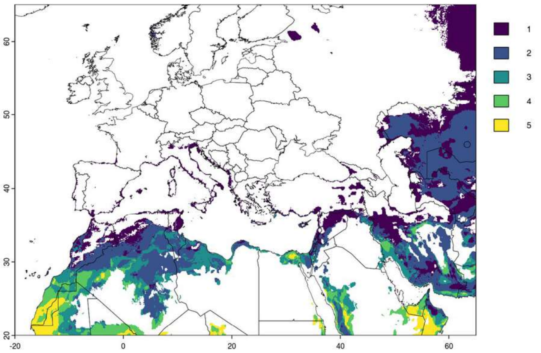

UKESM1-0-LL SSP5-8.5

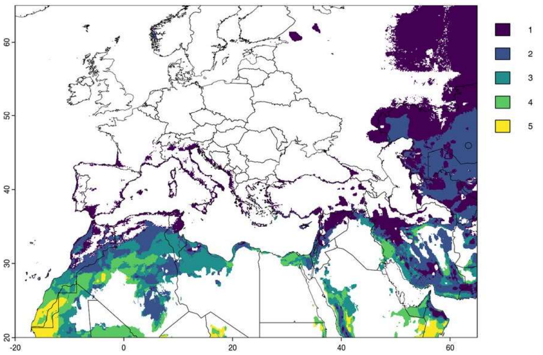

B) MOP for all variables representing where nonanalogous conditions were found towards high values of each variable

2011-2040

GFDL-ESM4 SSP3-7.0

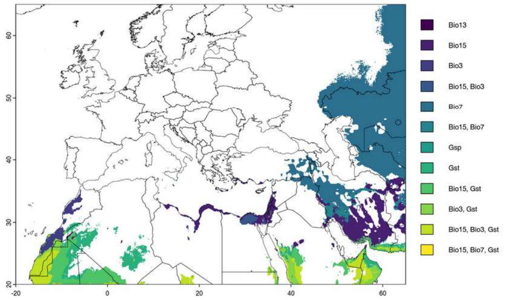

GFDL-ESM4 SSP5-8.5

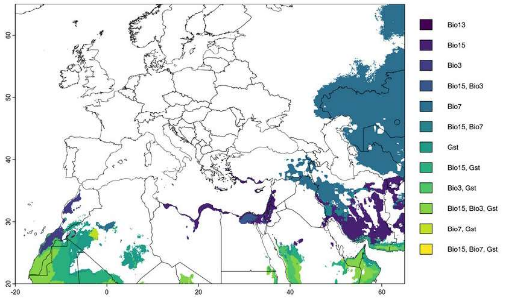

IPSL-CM6A-LR SSP3-7.0

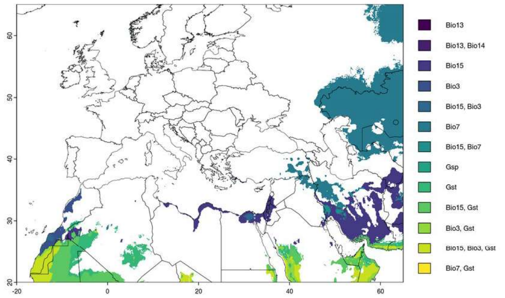

IPSL-CM6A-LR SSP5-8.5

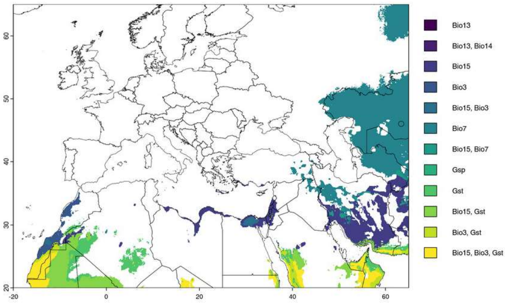

MPI-ESM1-2-HR SSP3-7.0

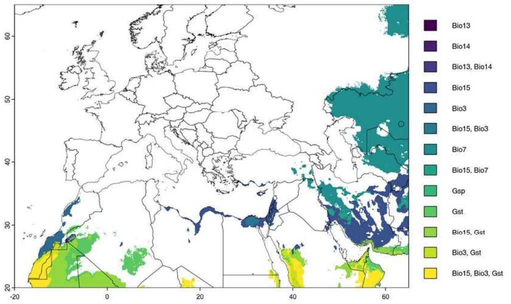

MPI-ESM1-2-HR SSP5-8.5

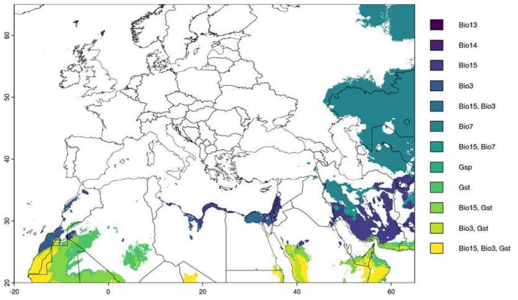

MRI-ESM2-0 SSP3-7.0

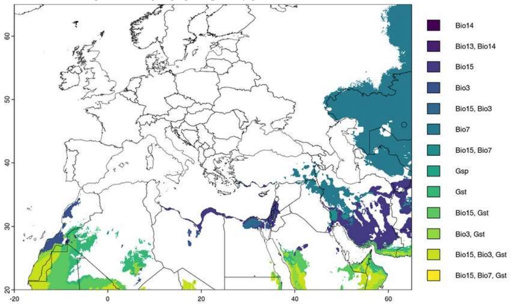

MRI-ESM2-0 SSP5-8.5

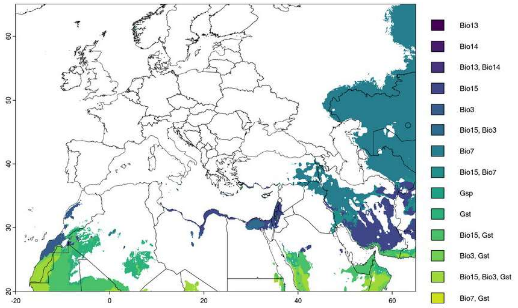

UKESM1-0-LL SSP3-7.0

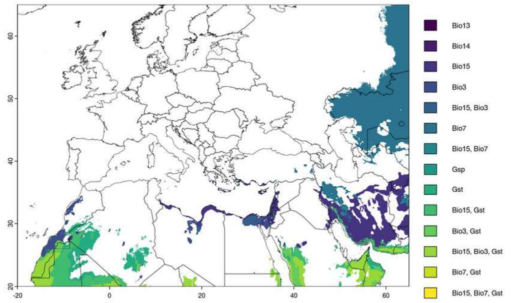

UKESM1-0-LL SSP5-8.5

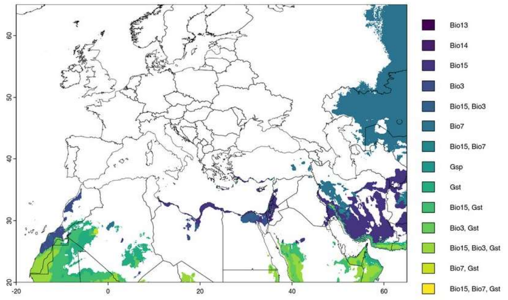

2041-2070

GFDL-ESM4 SSP3-7.0

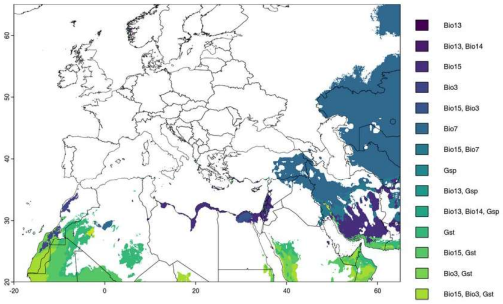

GFDL-ESM4 SSP5-8.5

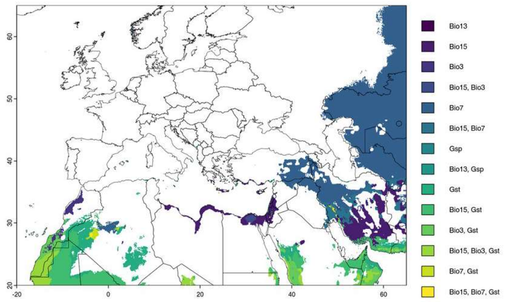

IPSL-CM6A-LR SSP3-7.0

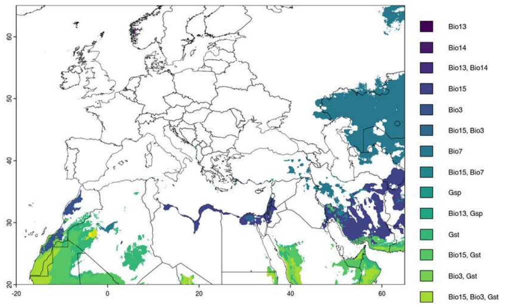

IPSL-CM6A-LR SSP5-8.5

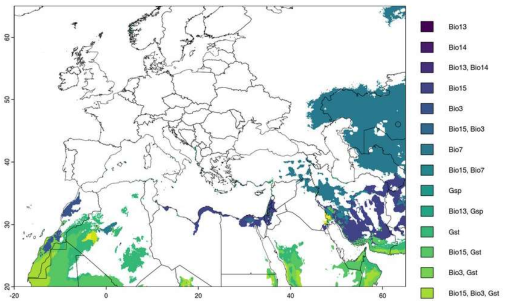

MPI-ESM1-2-HR SSP3-7.0

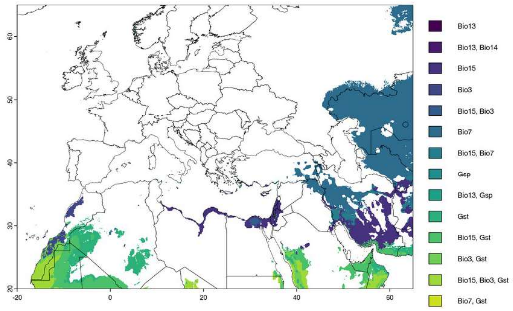

MPI-ESM1-2-HR SSP5-8.5

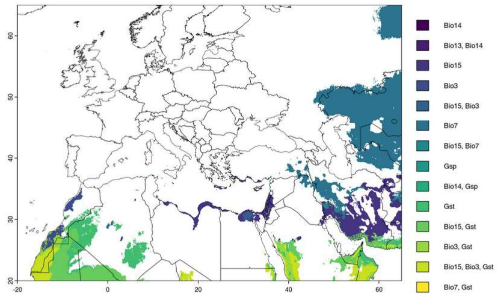

MRI-ESM2-0 SSP3-7.0

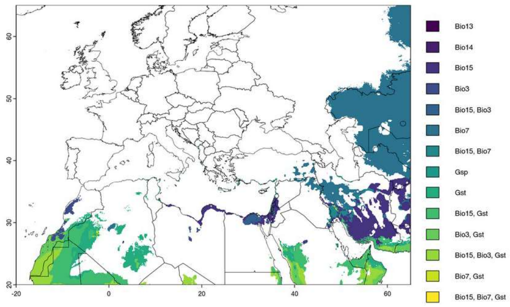

MRI-ESM2-0 SSP5-8.5

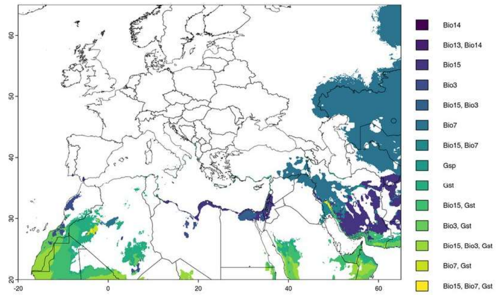

UKESM1-0-LL SSP3-7.0

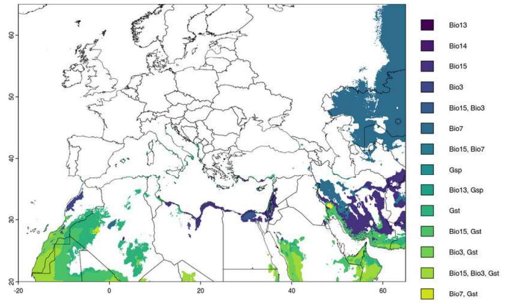

UKESM1-0-LL SSP5-8.5

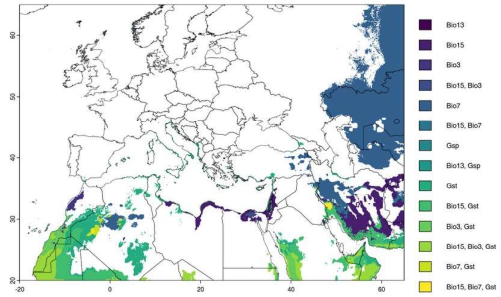

2071-2100

GFDL-ESM4 SSP3-7.0

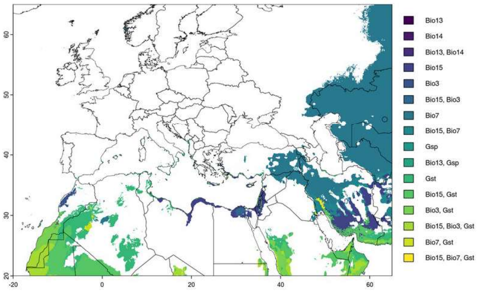

GFDL-ESM4 SSP5-8.5

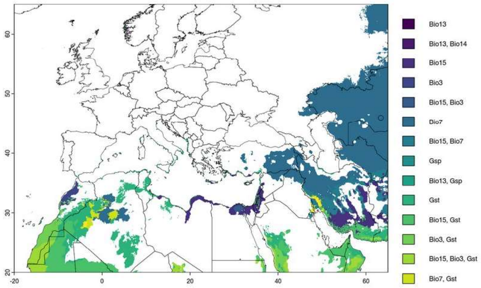

IPSL-CM6A-LR SSP3-7.0

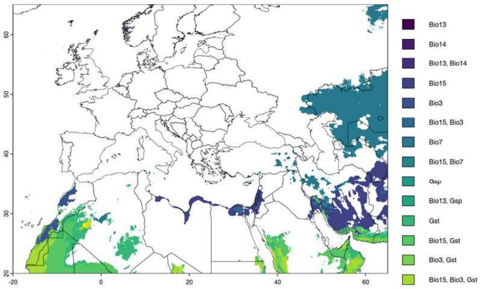

IPSL-CM6A-LR SSP5-8.5

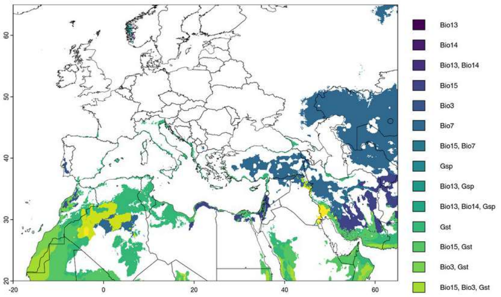

MPI-ESM1-2-HR SSP3-7.0

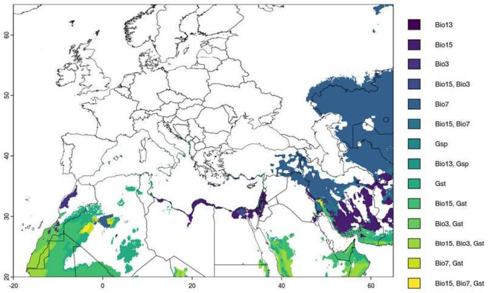

MPI-ESM1-2-HR SSP5-8.5

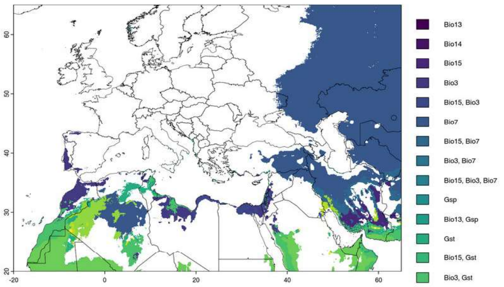

MRI-ESM2-0 SSP3-7.0

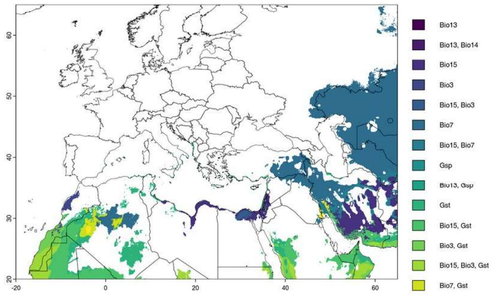

MRI-ESM2-0 SSP5-8.5

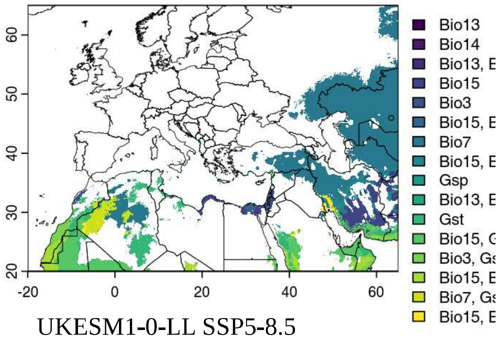

UKESM1-0-LL SSP3-7.0

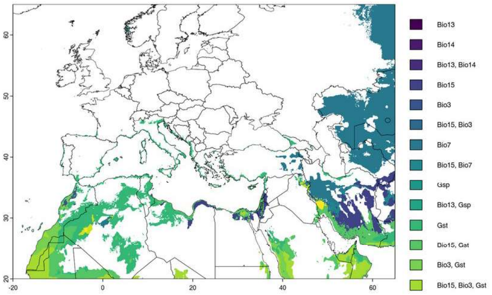

UKESM1-0-LL SSP5-8.5

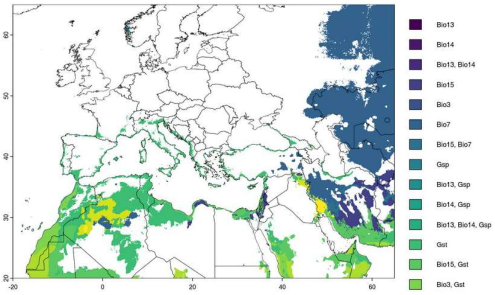

C) MOP for all variables representing where nonanalogous conditions were found towards low values of each variable

2011-2040

GFDL-ESM4 SSP3-7.0

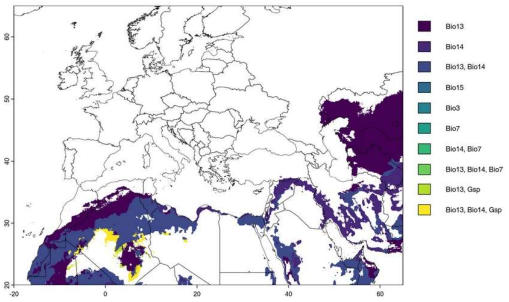

GFDL-ESM4 SSP5-8.5

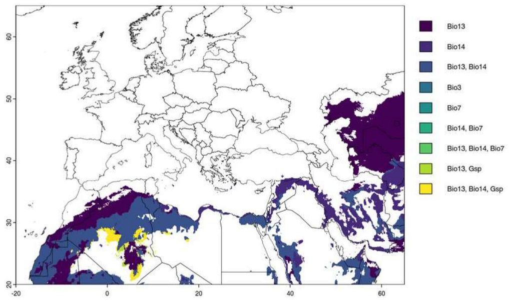

IPSL-CM6A-LR SSP3-7.0

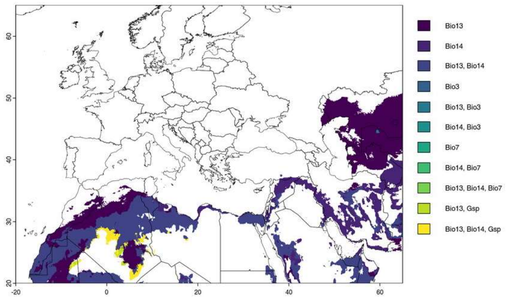

IPSL-CM6A-LR SSP5-8.5

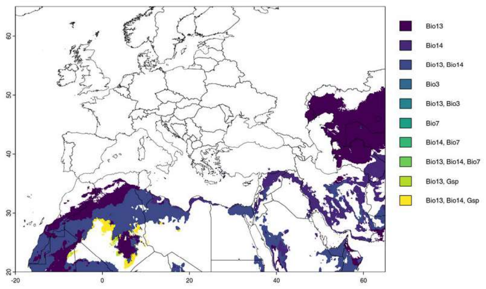

MPI-ESM1-2-HR SSP3-7.0

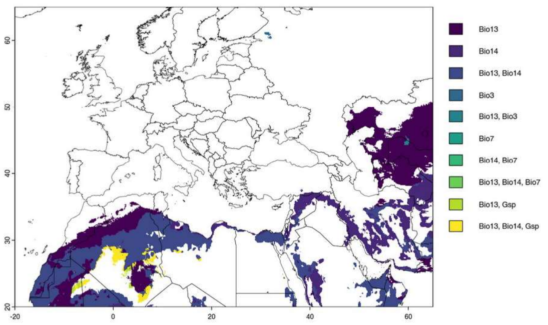

MPI-ESM1-2-HR SSP5-8.5

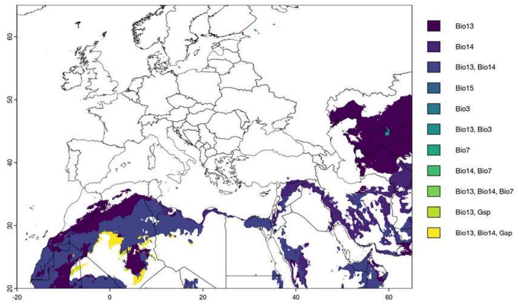

MRI-ESM2-0 SSP3-7.0

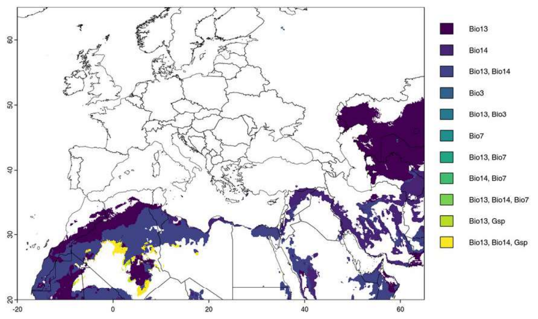

MRI-ESM2-0 SSP5-8.5

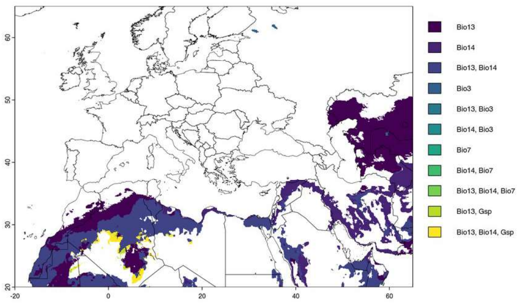

UKESM1-0-LL SSP3-7.0

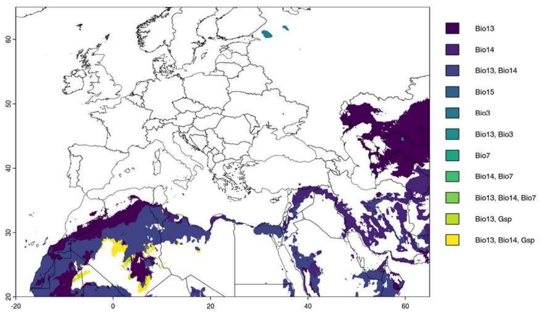

UKESM1-0-LL SSP5-8.5

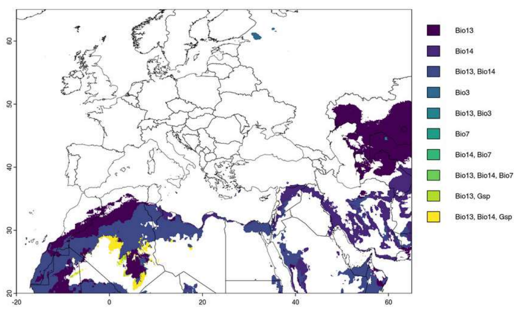

2041-2070

GFDL-ESM4 SSP3-7.0

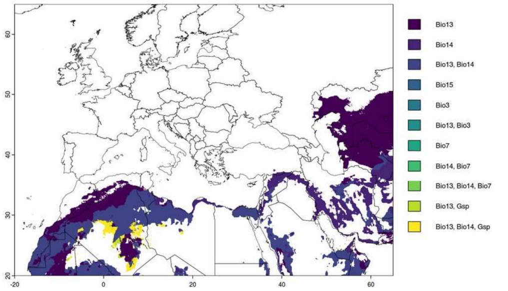

GFDL-ESM4 SSP5-8.5

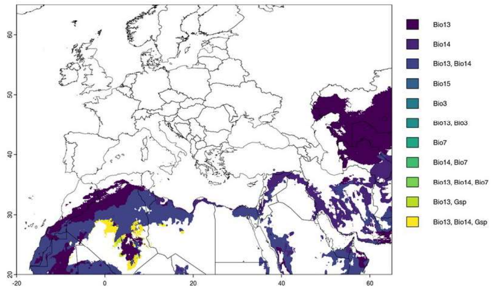

IPSL-CM6A-LR SSP3-7.0

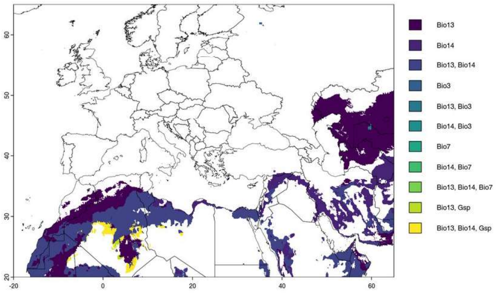

IPSL-CM6A-LR SSP5-8.5

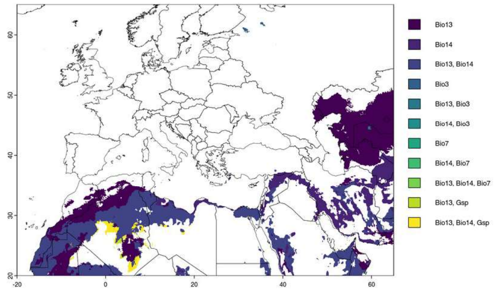

MPI-ESM1-2-HR SSP3-7.0

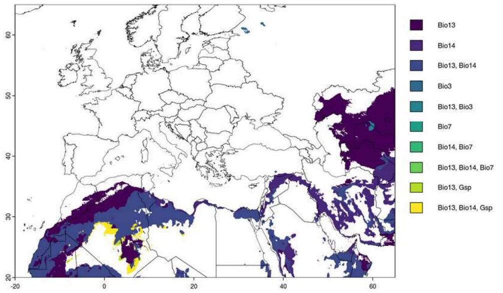

MPI-ESM1-2-HR SSP5-8.5

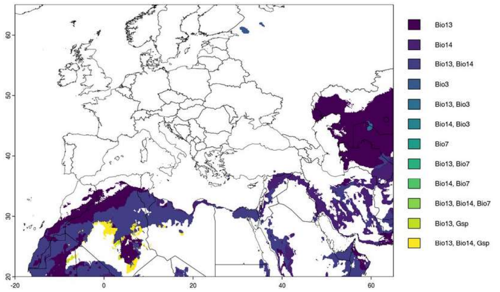

MRI-ESM2-0 SSP3-7.0

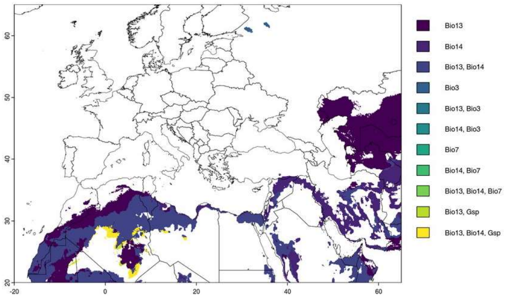

MRI-ESM2-0 SSP5-8.5

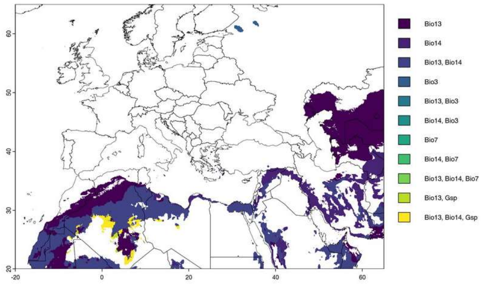

UKESM1-0-LL SSP3-7.0

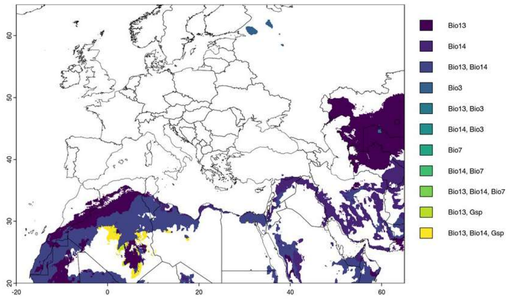

UKESM1-0-LL SSP5-8.5

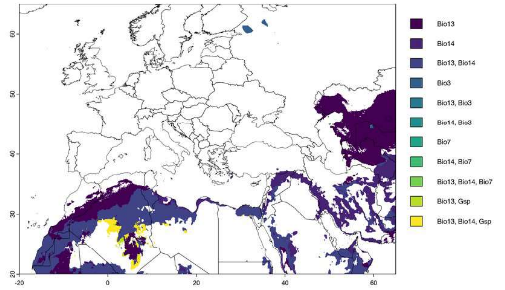

2071-2100

GFDL-ESM4 SSP3-7.0

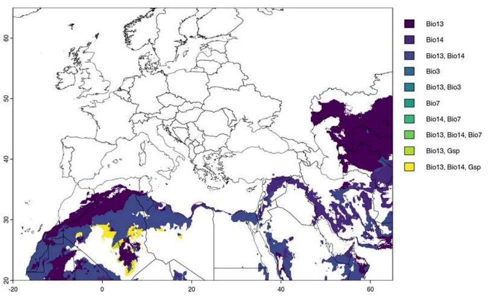

GFDL-ESM4 SSP5-8.5

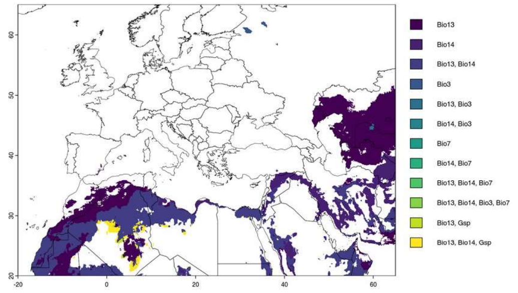

IPSL-CM6A-LR SSP3-7.0

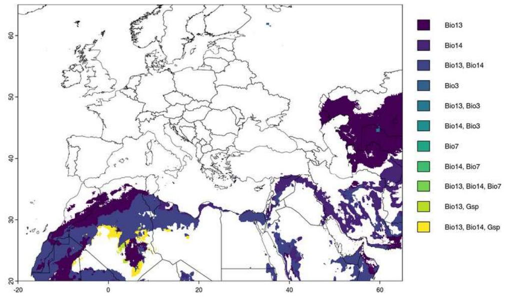

IPSL-CM6A-LR SSP5-8.5

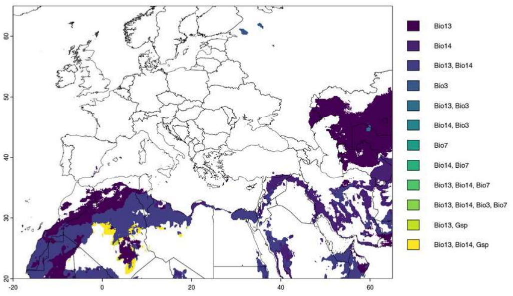

MPI-ESM1-2-HR SSP3-7.0

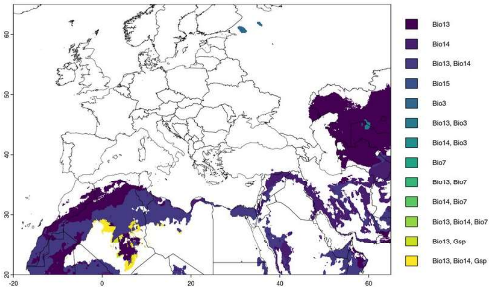

MPI-ESM1-2-HR SSP5-8.5

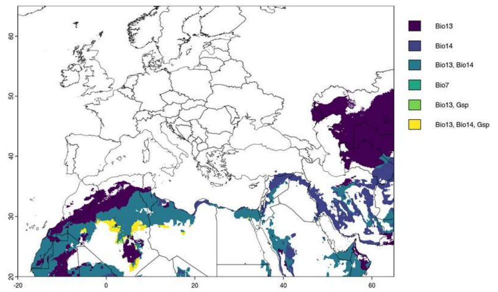

MRI-ESM2-0 SSP3-7.0

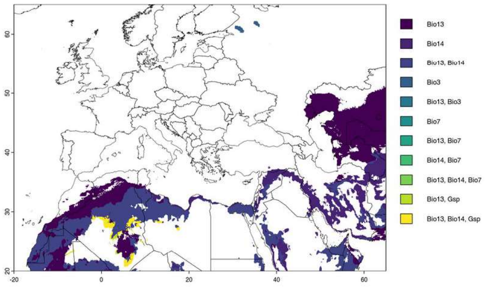

MRI-ESM2-0 SSP5-8.5

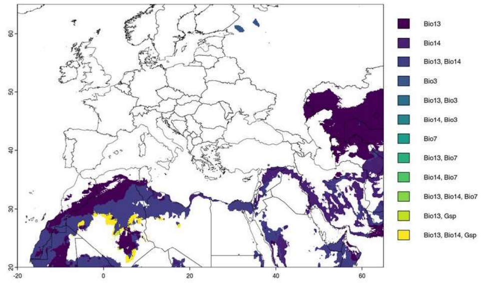

UKESM1-0-LL SSP3-7.0

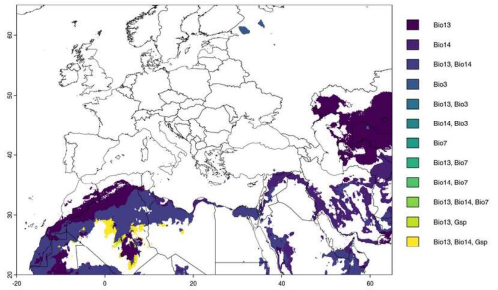

UKESM1-0-LL SSP5-8.5

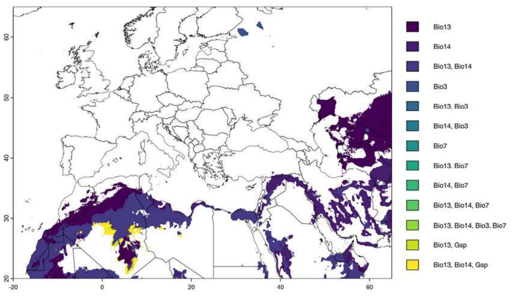

Supplement: Kuyucu and Hekimoglu supplementary material 2 — Kuyucu and Hekimoglu supplementary material [file S003118202400132Xsup002.pdf]
